# Linking the scaling of tremor and slow slip near Parkfield, CA

**DOI:** 10.1038/s41467-022-33158-3

**Published:** 2022-10-03

**Authors:** Hui Huang, Jessica C. Hawthorne

**Affiliations:** Department of Earth Sciences, University of Oxford, Oxford, UK

**Keywords:** Seismology, Geophysics

## Abstract

There has been much debate about the fault zone processes that generate slow earthquakes, including tremor and slow slip. Indeed, we still debate whether tremor and slow slip are generated by the same process operating at different scales or by two distinct processes. Here we investigate tremor scaling near Parkfield, California; we examine how rupture duration scales with moment. We thoroughly search for and detect the low frequency earthquakes (LFEs) that constitute tremor and robustly estimate their durations. Our results show varying durations (0.1–0.6 s) and spectra for LFEs at the same location. These variations confirm a common assumption, that LFEs’ observed low frequency contents are due to source processes, not path effects. The LFEs’ amplitude and spectra variations are consistent with a linear moment-duration scaling: the same scaling observed among slow slip events. The similar scaling suggests that tremor and slow slip events are governed by the same fault zone process and that when we attempt to identify the process creating slow earthquakes, we should focus on processes which allow higher slip rates on smaller faults.

## Abstract (web summary)

Huang and Hawthorne present new evidence supporting that tremor and slow slip are linked by the same moment-duration scaling. First-order implications would include that tremor and slow slip are likely generated by the same fault zone process.

## Introduction

We now know that many fault segments slip in slow earthquakes^1–10^. They slip in slow slip events (SSEs), when hundred-km-long fault segments accelerate but for some reason stall at ~100 times the plate rate^1–4^, as well as in tremor, when hundred-m-long segments repeatedly accelerate to ~10^6^ times the plate rate, creating low-frequency earthquakes (LFEs)^5–8^. Slow earthquakes can trigger or initiate large earthquakes^11–13^, therefore it is crucial to understand their underlying physical mechanisms. Tremor’s LFEs typically last ~0.2 s^14–17^: a brief period, but ~50 times longer than normal earthquakes of a similar size. We still do not know what happens in the fault zone during tremor and slow slip events. We do not know which process stops the fault from accelerating into faster, fully seismic ruptures^18–29^. Slip rates could be limited by a particular patch size^18,19^, by fault geometry^28^, by brittle-viscous shear^24,25^, or by shear-induced dilatancy^22,23^, for instance. Or multiple processes could be active. It is possible that one fault zone process arrests all slow earthquakes, but it is also possible that one process arrests tremor while another arrests slow slip.

**Fig. 1:**
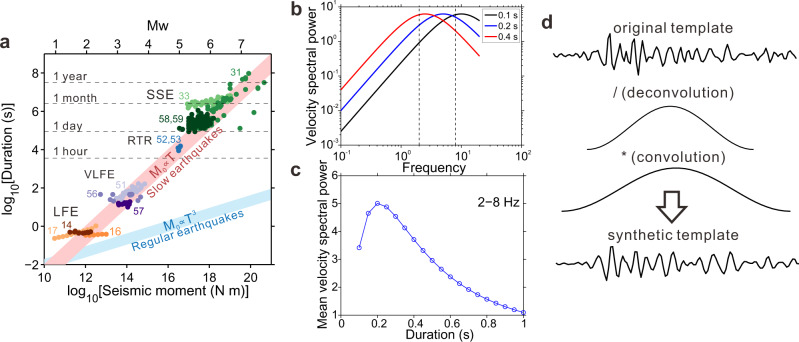
Previous slow-earthquake source observations and hypothesis of various-duration low frequency earthquakes. **a** Previous observations of moment and duration of slow earthquakes (colored circles) at a wide range of sizes^14,16,17,31,33,51–53,56–59^. LFE low-frequency earthquake, VLFE very low-frequency earthquake, RTR rapid tremor reversal, SSE slow slip event. **b** Theoretical Brune-type^55^ velocity spectral power for LFEs assuming a linear moment-duration scaling and a high-frequency spectral fall-off rate *γ* of 2 (see Methods). Note that this assumption is supported by our spectral observations (Fig. 5). Dashed lines (2–8 Hz) are the primary observation band of LFEs. **c** The mean spectral power between 2 and 8 Hz band for LFEs with different durations, after convolving with the 2–8 Hz Butterworth filter used in the detection. **d** shows how we create synthetic templates with different durations. We start with an original template built from stacked detections and deconvolve an assumed source time function. Then we convolve with source time functions of different durations.

Some researchers have suggested that a wide variety of slow earthquakes, including slow slip and tremor, are manifestations of the same process. They noted that these events’ wide-ranging sizes and durations fall along a systematic trend, where moment *M*_*0*_ scales linearly with duration *T* (*M*_*0*_ ∝ *T*, Fig. 1a)^9,30,31^. However, other researchers have examined slow-earthquake moments and durations by themselves and identified different scalings. Some found that LFE durations are independent of moment: that most durations are around 0.2 s near Parkfield^15^, 0.5 s in Cascadia^14^, and 0.3 s in Mexico^16^. And others have found that LFE or SSE durations scale as *M*_*0*_^1/3^ (*M*_*0*_ ∝ *T*^3^), following the same pattern as regular earthquakes^17,32–34^. These deviations from slow earthquake’s linear moment-duration scaling could indicate that LFEs and SSEs are created by different fault zone processes.

We note, however, that observations of a characteristic, moment-independent duration can in principle result from detection bias. LFEs are usually detected within a 2–8-Hz frequency band^5–8,14,35^, between low-frequency microseism noise and high-frequency local noise. That frequency band is well suited to detecting 0.2-sec-long LFEs, whose energy is concentrated in a band around 5 Hz (blue curve in Fig. 1b). Shorter and longer LFEs have energies that peak at higher and lower frequencies, respectively (black and red curves in Fig. 1b). If we assume that LFEs follow the linear moment duration scaling inferred for larger slow earthquakes, we can estimate spectral power for these shorter and longer LFEs. When we assume a high-frequency spectral fall-off rate *γ* of 2 (see Methods for definition of spectra, Fig. 1c and Supplementary Fig. 1), we find that 0.2-sec-long events should be the easiest events to detect; they should have larger average power than shorter and longer events in the 2–8-Hz band.

Here, we hypothesize that LFEs with a range of durations do exist: that we just need a more thorough approach to identify them. So we create synthetic LFEs with various durations. We search through the seismic data to find signals similar to these synthetics. Then we examine the durations, amplitudes, and spectra of the detected events and discuss their implications for slow earthquake processes.

## Results

### Detection and duration classification of LFEs

**Fig. 2:**
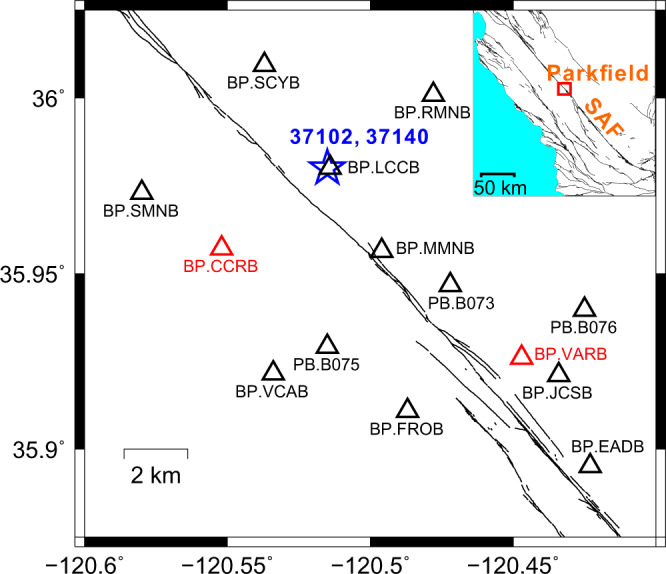
Study area and locations of seismic stations and templates. Map showing locations of the template events for two families (overlapping blue stars) and 14 seismic stations (triangles) in the Parkfield area. Red triangles denote two independent stations that are not used in matched-filter detection but are reserved for validations. Thin black lines denote the San Andreas Fault Zone (SAF), and the inset illustrates the larger-scale tectonics, with the red rectangle indicating the study area.

We search for and analyze LFEs along the San Andreas Fault Zone in California, where tremor has been studied extensively^36–41^, and where Shelly^7^ has identified over a million LFEs from 2001 to 2016. Based on waveform similarity, Shelly^7^ grouped the LFEs into 88 families, which represent slip on different patches of the fault. We investigate two LFE families (IDs: 37102 and 37140) along the Parkfield section, where local broadband seismic stations provide good coverage (Fig. 2). The average source durations for both families were estimated to be ~0.2 s via an empirical Green’s function method^15^. So we assume that the average seismograms created by LFEs in families 37102 and 37140 represent the shaking produced by a rupture that lasts 0.2 s. We, therefore, create template waveforms for 0.2-sec ruptures for both families by stacking the seismograms recorded at the times of Shelly’s detections^7^ (see Methods).

Next, we create template waveforms for events with different durations. We deconvolve the 0.2-sec templates with a 0.2-sec-long Hann-window source time function and then convolve with a Hann-window source time function of a different length: between 0.1 and 0.6 s (Fig. 1d). Once we have the templates, we filter the templates and the continuous seismic data to 2–8 Hz, and then we search for signals similar to the templates during ~850 days of continuous seismic data (see Methods), using a matched-filter detection algorithm^35,42^. We average the cross-correlation coefficients (XCCs) over the three components and over 12 out of 14 seismic stations. Stations CCRB and VARB are not used in the detection (Fig. 2 and Supplementary Fig. 2); they will serve as independent datasets for validating the detections’ durations and amplitudes.

**Fig. 3:**
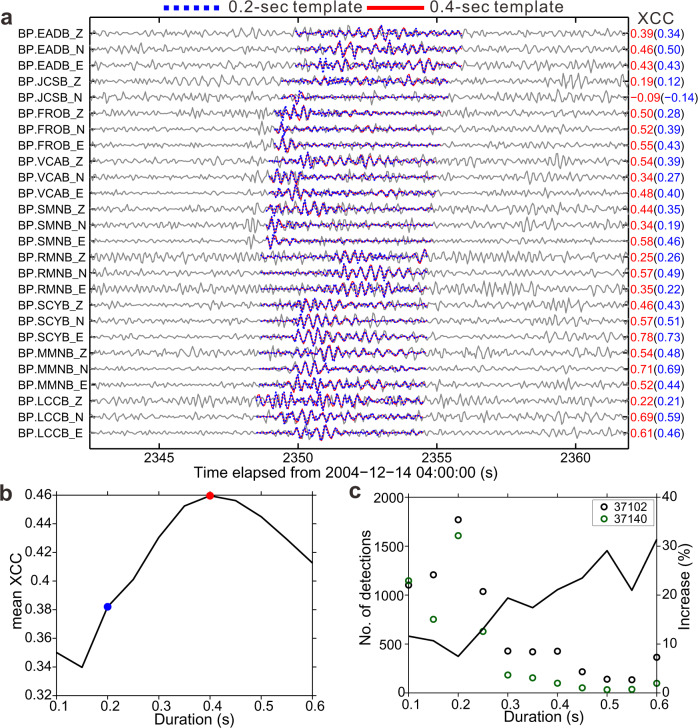
Detection and duration classification of low-frequency earthquakes. **a** Example of detection and duration classification of a low-frequency earthquake. Its duration is assigned to be 0.4 s. The blue and red waveforms are original and synthetic templates, with a duration of 0.2 and 0.4 s, respectively. The station codes and channels (E: BP2/SP2; N: BP3/SP3; Z: BP1/SP1) are labeled on the left while the individual cross-correlation coefficients (XCCs) between templates and detections are on the right. **b** Mean XCC over all channels as a function of duration for detection in **a**. Blue and red dots correspond to 0.2- and 0.4-sec templates, respectively. **c** Circles (left axis) show the number of detections at each duration for two families. The black curve (right axis) shows the increase of number of events at each duration, compared to the existing events in Shelly’s catalog^7^.

Figure 3a illustrates an example detection, where each duration’s template is cross-correlated with the continuous data. A detection is accepted when the average XCC exceeds a threshold chosen relative to the daily noise level (see Methods). The duration of the detection is given by the template yielding the highest average XCC. Figure 3a shows that for the detection shown, the 0.4-sec template has higher XCC than the 0.2-sec template at many individual channels (red and blue numbers). Figure 3b shows that the 0.4-sec template has higher station-averaged XCC than any other template. The duration of this detection is thus assigned to be 0.4 s. It is interesting to note here that template waveforms used in our study are highly similar, and the LFE signals are rarely much larger than the noise. However, synthetic tests with comparable noise levels and number of channels suggest that the changes in event durations are resolvable when we average XCC over multiple channels: the durations are misclassified only ~2% of the time (see Methods, Supplementary Fig. 3).

We detect 12,031 LFEs in the ~850 days of data analyzed. Each one is assigned a best-matching duration. Most of the detections were already in Shelly’s catalog^7^, and most of the detections have best-matching durations of 0.2 s. However, 18% of the detected LFEs have durations of 0.1 s, and 23% have durations of 0.3 s or longer. Longer LFEs are less likely to be in Shelly’s catalog^7^ (Fig. 3c). To further explore the detectability of LFEs with a range of durations, we also redo our analysis after filtering the data to different frequency bands: 2–4 and 4–8 Hz. The ratio of long events to short events increases when we focus on lower frequencies (Supplementary Fig. 4). It would appear that low frequencies facilitate the detection of longer LFEs, which have energy concentrated at low frequencies (red curve in Fig. 1b), while higher frequencies facilitate the detection of shorter events, which have energy concentrated at higher frequencies (black curve in Fig. 1b).

**Fig. 4:**
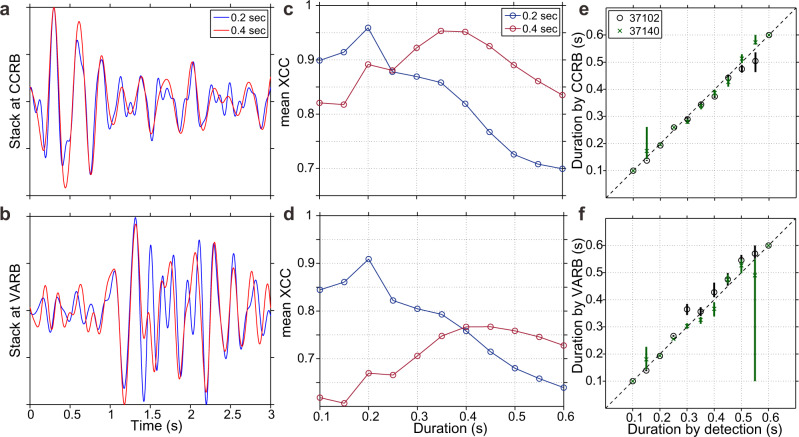
Validating durations of low-frequency earthquakes at independent datasets. **a**, **b** Waveforms from stacking 0.2-sec (blue) and 0.4-sec (red) detections (component E) at station CCRB and VARB: stations that were not used in the detection. **c**, **d** Mean cross-correlation coefficients (XCCs) between the three-component stacks and different-duration synthetic templates at station CCRB and VARB, respectively. At both stations, the 0.2-sec stack best matches the 0.2-sec template (blue), and the 0.4-sec stack best matches the 0.4-sec template (red). **e**, **f** Comparison between the durations estimated by 12 stations used in the detection and those estimated by station CCRB and VARB, respectively. The dashed lines mean a perfect match. The confidence bounds are 15th and 85th percentiles estimated from bootstrapping (see Methods). Some error bars cannot be clearly seen because they are too small.

We would like to interpret our 0.1, 0.2, and 0.4-sec detections as LFEs with a range of durations. Before we do so, however, we must check that the durations are reliable: that we are not simply finding signals similar to the templates amongst random noise. To do so, we first stack the waveforms of LFE detections at independent stations CCRB and VARB (Fig. 2), which were not used in the matched-filter detection. Figure 4a, b show the CCRB and VARB stacks after averaging the waveforms of 0.2-sec detections (blue) and the waveforms of 0.4-sec detections (red). The 0.2-sec and 0.4-sec stacks differ subtly. By cross-correlating these stacks with the corresponding synthetic templates at CCRB or VARB separately, we find that stacks from 0.2-sec detections most resemble a 0.2-sec template while 0.4-sec stacks most resemble a 0.4-sec template (Fig. 4c, d). Similar consistency is found for stacks with other durations (Fig. 4e, f). We further estimate the uncertainty of the CCRB and VARB durations by bootstrapping the detections included in the stacks (see Methods). The 70% confidence bounds on the stacks’ durations are mostly smaller than 0.05 s (sampling interval, Fig. 4e, f). These small uncertainties imply that each duration group might contain a small portion of LFEs which were assigned the wrong duration, but those misclassified LFEs do not appear to significantly bias the stack’s characteristics.

To further check that our durations are reliable, we also examine our detections’ multi-taper spectra^43^. Our motivation here is to avoid alignment-induced bias. We have obtained longer, smoother waveforms by stacking long-duration detections. But we want to rule out the potential bias that longer, smoother waveforms could result from stacking poorly aligned short events. Such a bias should not arise with spectra; the energy in a given window varies minimally with a 0.1-sec error in detection time. We compute the median spectral power in groups of the detection intervals. We isolate the signal power from the noise by subtracting the noise spectral power in windows that precede the detections. Then we obtain the median noise-corrected spectral power for detections in each duration group. This averaging allows us to recover the signal power even though the noise power can be larger than the signal power during many LFE signals (see Methods and Supplementary Fig. 5). We find that at most stations, the averaged spectral power of longer-duration detections have more of their energy concentrated at low frequencies (Supplementary Fig. 6). Such a shift in energy is expected for longer-duration ruptures (Fig. 1b); the shift to low frequencies would not result from misaligned short ruptures.

### Evidence for linear moment-duration scaling

**Fig. 5:**
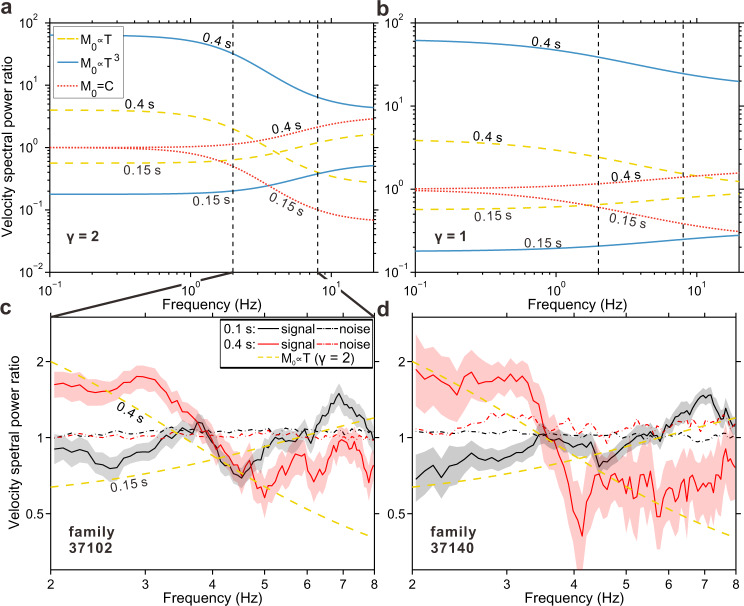
Evidence for linear moment-duration scaling from spectral observations of low frequency earthquakes. **a**, **b** show the theoretical velocity spectral power ratios for different moment-duration scalings, assuming a high-frequency spectral fall-off rate *γ* of 2 and 1, respectively (see Methods). *M*_*0*_ = *C* means that the moment is constant, independent of duration. **c**, **d** Black and red solid lines show the median noise-corrected spectral power (component E) observed at the times of 0.1-sec and 0.4-sec detections, as normalized by the median power observed at times of 0.2-sec detections. The gray and pink shaded confidence regions show the 15th and 85th percentiles estimated from bootstrapping (see Methods). Yellow dashed lines in **c**, **d** are the same as those in **a**. Dashed-dotted lines show the median power observed in noise windows that precede the 0.1-sec and 0.4-sec detections, as normalized by the median power in noise windows preceding 0.2-sec detections (Supplementary Fig. 7).

The inferred durations and spectra of the grouped durations suggest that we have successfully identified groups of LFEs with different durations. We will use the groups’ spectra and then the groups’ waveform stacks to probe the scaling between LFE moment and duration. First, however, we must note that the stacked spectra include site and path effects, which we do not know. So instead of examining the spectra directly, we examine spectral ratios^44–46^, which divide out the site and path effects. At each station, we divide the median noise-corrected spectral power from 0.1- and 0.4-sec detections by the median power from 0.2-sec detections. Then we take the median among stations to obtain 0.1- and 0.4-sec spectral power ratios (see Methods for details). For example, in Fig. 5c, d, the plotted ratios show that the 0.4-sec spectra are larger than the 0.2-sec spectra at low frequencies but become smaller than the 0.2-sec spectra at high frequencies. In contrast, the 0.1-sec spectra are smaller than the 0.2-sec spectra at low frequencies but higher than the 0.2-sec spectra at high frequencies. We estimate uncertainties on the spectral power ratios by bootstrapping the detections included in each average (shaded regions in Fig. 5c, d, see Methods). The variation in frequency content among the different-duration groups is much larger than that uncertainty. For comparison, we also plot the noise spectral ratios, taken from intervals just before the detections (dashed-dotted curves in Fig. 5c, d). The noise spectral ratios are almost constant with frequency; long-duration LFEs do not appear associated with long-period noise. Having identified trends in the spectral ratios with frequency, we compare our observations to predictions from different moment-duration relations: to the cases where *M*_*0*_ ∝ *T* (yellow in Fig. 5a, b), *M*_*0*_ ∝ *T*^3^ (blue), and *M*_*0*_ *=* *C* (constant, *M*_*0*_ is independent of *T*, in red). In panel (a), we assume that the high-frequency spectral amplitude decays as *f*^*−2*^ (*γ* = 2, *f* is frequency, see Methods), and in panel (b), we assume that it decays as *f* ^*−1*^ (*γ* = 1). None of the predictions match the data perfectly, even when we have chosen to model the 0.1-sec observed spectra with a 0.15-sec prediction to allow a better match. Our detection approach is better suited and validated for longer-duration detections. But the prediction that best matches is the one with *M*_*0*_ ∝ *T* and *γ* = 2 (yellow in Fig. 5a). Only *M*_*0*_ ∝ *T* with *γ* = 2 allows the 0.15-sec and 0.4-sec (or 0.1-sec and 0.4-sec, not shown) spectral ratios to cross, as seen in our observations.

**Fig. 6:**
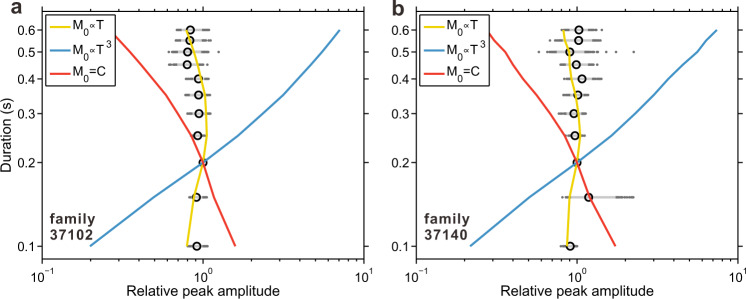
Evidence for linear moment-duration scaling from amplitude observations of low-frequency earthquakes. **a**, **b** Black circles denote the observations: duration vs amplitude for stacks of LFEs in family 37102 and 37140 (component E), respectively. The amplitudes are normalized by the amplitude of the 0.2-sec stack. The gray dots denote the measurements from bootstrapping the traces 500 times during stacking, while the light gray bars denote the 5th and 95th percentiles. The yellow, blue, and red solid curves show the amplitude variations predicted from various moment-duration scalings (see Methods), as labeled. ${M}_{0}{{\mbox{=}}}C$ means that the moment is constant, independent of duration.

We further probe the LFEs’ moment-duration scaling by examining the amplitudes of each group of detections. The data are noisy, so it is not practical to determine amplitudes of individual LFEs. So instead, we stack waveforms for detections at each duration without any normalization. We isolate high-quality stacks, those with signal-to-noise ratios larger than 5, and measure their peak absolute amplitudes. We normalize the amplitudes by the amplitude of the 0.2-sec stack at each station and then take the median of all amplitudes. The obtained amplitudes are plotted as black circles in Fig. 6a, b. We find roughly duration-independent amplitudes, and those amplitudes do not appear strongly biased by detection capability; we obtain similar amplitude ratios when we redo our analysis in 2–4 Hz and 4–8 Hz frequency bands (Supplementary Figs. 8, 9) or at independent stations CCRB and VARB, albeit with larger uncertainty (Supplementary Fig. 10).

We compare the observed amplitudes with those predicted for different moment-duration scalings (colored lines, Fig. 6a, b, see Methods). The result shows that the observed amplitudes agree well with the predictions from the linear moment-duration scaling, where *M*_*0*_ ∝ *T*, but are inconsistent with other scaling relations (Fig. 6). The consistency with only the linear moment-duration scaling persists as we account for detection bias. In the supplementary material, we carry out synthetic tests to see how the detectability of longer and shorter LFEs could influence our results, but we do not find another moment distribution or moment-duration scaling that could match our stacks’ amplitudes (see Supplementary Text and Supplementary Figs. 12–19).

## Discussion

We have detected, stacked, and examined the spectra of groups of LFEs at two locations along the San Andreas Fault. The identified groups of LFEs display a range of durations, and those durations vary linearly with moment. Our approach relies on stacking, so we can resolve only the average behavior of each group, not the individual LFE source properties. Nevertheless, the average trends are revealing; the LFEs’ durations and moment-duration scalings have first-order implications for tremor detection approaches and for tremor source mechanisms.

Our detection approach is able to find more long (~0.4 s) LFEs than previous studies in Parkfield^7^. These long-LFE detections highlight the importance of thoroughly searching for small events hidden in the data. We do preferentially detect different-duration events when we filter to different frequency bands, as one might expect given the amplitudes and frequency contents of LFEs that follow a linear moment-duration scaling (Fig. 1b). These frequency-dependent detections suggest that previous studies could miss longer or shorter events: that the apparently characteristic duration and frequency noted by some studies^14–16,47^ could reflect observational capability and thresholding, not a physical property of tremor. Furthermore, even when LFEs with various durations are detected, the data uncertainty might be large so that the scaling is not well constrained; for example in the supplement, we reanalyze a published dataset^17^ and show how uncertainties could introduce bias in the inferred scaling (Supplementary Text and Supplementary Figs. 20–22).

On the whole, however, the varying durations and frequency contents of our LFEs should be reassuring to tremor source studies. They confirm that LFEs’ low-frequency seismograms^5–9^ reflect the durations of LFE ruptures. The varying frequency content cannot be created by a region at depth that attenuates high-frequency seismic waves^48–50^, as that region would attenuate all LFEs in a given location in the same way.

We can, therefore, go further and examine how LFEs’ slip rate, stress drop, and duration could scale with rupture area. Our data imply that LFEs follow a linear moment-duration scaling, similar to that inferred for larger slow earthquakes^9,30,31^. From basic seismology, we know that moment is proportional to the area of the LFE ruptured patch times the average slip on that patch. Thus we have *M*_*0*_ ∝ *AD = AV*_*slip*_ *T*, where *A* is rupture area, *D* is slip and *V*_*slip*_ is the average slip rate. Given the observed scaling, *M*_*0*_ ∝ *T*, we have *AV*_*slip*_ *T* ∝ *T*, or *V*_*slip*_ ∝ *A*^−*1*^. This implication of the linear moment-duration scaling—that slip rates are inversely proportional to rupture areas—has long been recognized as surprising and revealing. It suggests that smaller ruptures, despite releasing less energy, somehow slip faster.

**Fig. 7:**
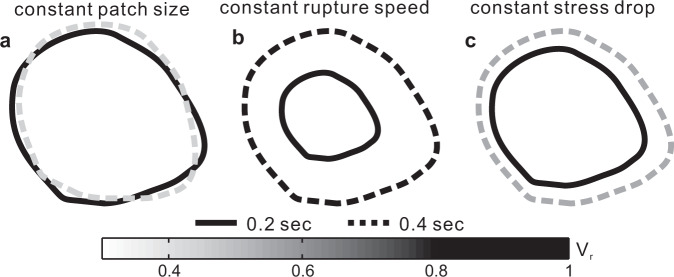
Possible rupture scenarios of low-frequency earthquakes. Three possible scenarios for low-frequency earthquakes evolving from duration of 0.2 s (solid curve) to 0.4 s (dashed curve). Low-frequency earthquakes of 0.4 s are slow ruptures of the 0.2-sec patch (**a**), low stress drop ruptures of a much larger patch (**b**), or slow ruptures of a partially adjacent patch (**c**). Scenario (**c**) could be viewed as a combination of **a**, **b**. Colors of boundaries indicate rupture speed (*V*_*r*_).

Further constraints on LFE rupture properties are limited. We may note that moment scales as $\varDelta \tau {V}_{r}^{\,3}{T}^{3}$, where $\varDelta \tau$ is the stress drop on the patch and *V*_*r*_ is the rupture speed. So in order for the moment to scale linearly with duration *T*, larger LFEs must rupture more slowly or have lower stress drops. And we may speculate about possible LFE rupture scenarios^9,14,28,47^, as illustrated in Fig. 7. Larger, longer LFEs (gray) could arise when (a) a similar patch ruptures with a higher stress drop, a lower rupture speed, and the same slip rate, when (b) a much larger patch ruptures with a lower stress drop, a similar rupture speed, and a lower slip rate, or when (c) a larger patch ruptures with a similar stress drop, a lower rupture speed, and a lower slip rate. One could speculate about a wide range of mechanisms to create such changes in velocity and stress drop within the LFE duration band: from 0.1 to 0.6 s.

However, a linear moment-duration scaling (pink band in Fig. 1a) tracks most observed slow earthquakes over a wide range of sizes: from our 0.2-sec-long LFEs to our 0.4-sec-long LFEs, and then to 30-sec-long VLFEs, 3-h-long rapid tremor reversals, and 3-week-long slow slip events (Fig. 1a)^9,30,31,51–53^. This simple scaling suggests that LFEs and slow slip events are generated by the same fault zone process: that LFEs are short slow slip events. With this hypothesis, we must discard constant-patch (Fig. 7a) and constant-speed (Fig. 7b) rupture scenarios, as slow slip events rupture larger areas much more slowly than LFEs^1–4,15,17,47,54^. The constant-stress-drop scenario (Fig. 7c), on the other hand, could accommodate slow slip events’ larger rupture areas and lower slip rates. This is somewhat consistent with observations showing that stress drops inferred for LFEs are similar to those inferred for slow slip events^15,31^.

The above LFE rupture scenarios are very simplistic hypotheses: the relations between source parameters are highly nonlinear, and the realistic ruptures are likely more complex and heterogeneous. More robust LFE source observations are required to differentiate them. The key implication of the linear moment duration scaling is that ${V}_{{slip}}\propto {A}^{-1}$: that slip rates are larger in smaller slow earthquakes. As pointed out by Ide et al.^9^, the variation in slip rate is dramatic: from 10^−7^ m/s in slow slip events to 10^−3^ m/s in LFEs. These size-dependent slip rates suggest that the fault zone processes that create LFEs and slow slip events should limit slip rate growth more strongly on larger fault segments than on smaller fault segments. This inference could place strong constraints on our ongoing search for the fault zone processes^18–29^ that limit slow earthquake slip rates, as only a few of the proposed mechanisms could create size-dependent slip rates. For instance, we could exclude models with temperature-dependent slip rates, such as minimum asperity sizes^20^ and chemical reactions^27^, as the temperature is unlikely to vary systematically with fault size. We would favor processes like dilatational strengthening^22,23^, where slip rate could depend on fault zone width, or brittle-viscous shear^24,25^, where slip rate could depend on the density of brittle asperities.

Our observed LFEs provide one more piece of the slow earthquake spectrum. However, there are still gaps and discrepancies in the spectrum. More consistent and systematic analyses are needed to understand which gaps are due to observational limits and whether there is a single fault zone process that produces a continuum of slip rates.

## Methods

### Theoretical LFE spectra

We compute theoretical Brune-type velocity amplitude spectra^55^ for LFEs:

1 $$V\left(f\right)=2\pi C{M}_{0}f/(1\,+\,{(f/{f}_{c})}^{\gamma })$$

where *V(f)* is velocity spectral amplitude at frequency *f*, *C* is a constant, *M*_*0*_ is moment, *f*_*c*_ is the corner frequency and *γ* is the high-frequency spectral fall-off rate. Velocity spectral power is then *V*^*2*^*(f)*. Duration of an LFE (*T*), defined as the width of the hann-window function in this study, corresponds to the inverse of corner frequency (*T* = 1/*f*_*c*_). Then we calculate the theoretical velocity spectral power for different *T* and *γ*, compare the average power in the 2–8 Hz band (Fig. 1b, c and Supplementary Fig. 1) and compare the spectral power-ratio predictions from different scalings to the observations (Fig. 5). We can omit the constant 2*πC* in calculation as we are only interested in the relative spectral power.

### Data processing details for LFE detection and duration classification

To reduce computational costs, we analyze data recorded in selected days from Shelly (2017)’s catalog^7^. We sort the daily number of LFEs within families 37102 and 37140 (from 2003 to 2016). We select those most active 858 days when > 95% of LFEs occur. We collect continuous seismic data recorded by 14 stations (Fig. 2), operated by the High-Resolution Seismic Network (HRSN, network code: BP) and Plate Boundary Observatory (network code: PB) networks (Fig. 2). After removing the mean and trend from the data, a two-way fourth order 2–8 Hz Butterworth filter is applied to improve the signal-to-noise ratios of tremors. Then the data are resampled to 20 Hz if needed. We build template waveforms for families 37102 and 37140 from stacking all detection windows (20 s) centered at detection times for each event in the catalog of Shelly^7^. Then a series of windows (6-sec long) are moved through stacked channels (20-sec long) across all stations, with a step of 0.05 s. Starting times of windows follow the theoretical S-wave move out. The 6-sec template window is found at the position where total S-wave energy maximizes. The resulting template waveforms for families 37102 and 37140 are shown in Supplementary Fig. 2a,b, respectively. They represent the original template waveforms (0.2-sec duration) in our template dataset.

In the next step, the original template waveforms are modified into a series of synthetic templates with durations of 0.1–0.6 s (see Fig. 1d and main text), with a step of 0.05 s. Then templates with all durations are scanned through the same continuous data to detect events. Note that during the detection, templates at two independent stations (red triangles in Fig. 2; red traces in Supplementary Fig. 2a,b) are not used but reserved for validation of detection (see main text). For each day, template waveforms are cross-correlated with the corresponding continuous channels, moving through with a step of 0.05 s. We require at least 12 continuous channels for the analysis. Then all cross-correlation-coefficient traces are shifted according to the S-wave move out and averaged across all channels. To define the positive detection, a threshold of 11 times the median absolute deviation (MAD) of the trace for each day is defined separately^11,60^. There are frequent cases when the same event is detected by templates from different families or the same family with different durations. To remove the duplicate detections, only the one with the highest XCC is kept within the same 6-sec window. In this way, once an event is detected, it is also assigned with an optimal duration corresponding to the best-matching template.

We note that template waveforms used in this study are quite similar, for example, 0.2-sec and 0.4-sec template waveforms (component E) at CCRB have a XCC of ~0.86, while 0.2-sec and 0.3-sec template waveforms have a XCC of ~0.97. Thus noise could deteriorate the duration classification with single channel cross-correlation. Here, we carry out simple synthetic tests to compare the chance of duration misclassification between single- and multi-channel cross-correlation. For the multi-channel case, we use 25 channels, similar to the total number of channels used in the matched-filter detection. We also set the signal-to-noise ratios of synthetic data to be lower than that estimated from real data (Supplementary Figs. 6 and 7). We show that, with 25 channels, durations are misclassified only ~2% of the time, much lower than the chance of misclassification with a single channel (~33%, Supplementary Fig. 3).

In addition, we estimate our duration uncertainties at two independent stations: CCRB and VARB. For each duration group, we generate 500 samples of three-component stacks by bootstrapping the detection waveforms. Then for each sample, we estimate the duration at station CCRB and VARB, by cross-correlating the three-component stacks at CCRB and VARB with the corresponding various-duration three-component templates at CCRB and VARB, respectively, followed by averaging the three XCC at these two stations. We then obtain the average XCC as a function of duration. The optimal duration is estimated at subsample precision by fitting a parabolic curve to the largest XCC and its two neighbors. Note that when the maximum duration occurs at the edge, i.e., 0.1 or 0.6 s, it is impossible to do the parabolic fitting and the duration is simply kept as it is. Once we have all 500 duration estimates for each duration group, we estimate the 70% confidence bounds of duration by taking the 15th and 85th percentiles.

### Details on LFE spectra calculations

We calculate the noise-corrected spectral power for all LFEs, group the corrected spectral power by duration, and calculate the spectral power ratios. We first calculate the spectral power for each LFE detection window, then subtract the noise power calculated in an interval just before the detection:

2 $${PSDcorr}\_\,{{LFE}}_{i,j}={PSD}({data}_j[{t}_{i,j},{t}_{i,j}+6s])-{PSD}({data}_j[{t}_{i,j}-8s,{t}_{i,j}-2s])$$

where *PSDcorr_LFE*_*i,j*_ is the noise-corrected spectral power for LFE *i* at station *j*, *data*_*j*_ is the continuous data (component E) recorded at station *j*, *t*_*i,j*_ is the detection-window starting time for LFE *i* at station *j*. The spectral power, i.e., the power spectral density (PSD) is estimated by the multi-taper method^43^, using 5 tapers (*NW* = 3). This noise correction is based on the assumption that the noise power does not vary within 8 s. For each station, we group the noise-corrected spectra by duration (0.1, 0.2, or 0.4 s) and take the median spectra for each group. Then we divide the median 0.1-sec and 0.4-sec spectra by the median 0.2-sec spectra, to obtain the 0.1-sec and 0.4-sec spectral power ratios at each station. Finally, we take the median of the 0.1-sec and 0.4-sec spectral power ratios among all stations. To estimate the uncertainties of power ratios, for each duration group, we bootstrap the noise-corrected spectral power 500 times during stacking. Then we obtain the spectral power ratios for all 500 samples and estimate the 15th and 85th percentiles (Fig. 5c, d).

It is worth noting that, in our analysis, the average noise power is larger than the average LFE signal power (Supplementary Figs. 6 and 7). It is thus important to stack multiple event spectra for each duration group to recover the average LFE spectra. We carry out a simple synthetic test to show that, given the stacking of multiple noise-corrected spectral power, the input signal power can be reasonably well recovered even if the noise power is several times larger than the signal power (Supplementary Fig. 5). In the synthetic test, we process the noise correction and spectra stacking in the same way as the above LFE spectra analysis. Within the 2–8 Hz band, the signal-to-noise ratios in the synthetic test are comparable to or lower than those estimated from real data (Supplementary Figs. 6 and 7). The number of synthetic event spectra (500) is similar to the number of 0.4-sec events for family 37102. We also test the case of stacking 100 event spectra and still find that the input spectra can be well recovered (not shown).

### Mapping LFE moment into amplitude

We describe how we map an LFE’s moment into its amplitude. Note that moment and amplitude are ratios relative to the moment and amplitude of the 0.2-sec LFE, respectively. We first estimate the LFE amplitude as a function of duration, from the synthetic LFEs with the same moment. We generate synthetic LFEs with durations of 0.1–0.6 s, using the approach described in the main text. The convolved source time functions here are normalized by the area under the curve so that all synthetic LFEs have the same reference moment (*M*_*0ref*_ = 1). Then we measure the peak absolute amplitudes (component E) for all synthetic LFEs. At each station, we normalize the measured amplitudes at different durations by the 0.2-sec event amplitude. Then for each duration *T*, we take the median amplitude among stations (*A(T)*). It shows that *A(T)* decays approximately as the inverse of duration (Supplementary Fig. 11). For an arbitrary LFE with duration *T*_*0*_ and moment *M*_*0*_, we can obtain its amplitude *A*_*0*_ by:

3 $${A}_{0}({T}_{0})=({M}_{0}/{M}_{0{ref}})A({T}_{0})={M}_{0}A({T}_{0})$$

Finally, we generate moments and durations (0.1–0.6 s) following linear, cubic, and moment independent of duration scalings. In all cases, the moment at 0.2 s is set to 1. Then we map the moments at different durations into amplitudes, using the equation above. The resulting amplitude-duration relations are plotted as colored lines in Fig. 6 and elsewhere in the paper.

## Supplementary information

Supplementary information

Description of Additional Supplementary Information

Supplementary Data 1

## Data Availability

Seismic data used in this study were downloaded from the North California Earthquake Data Center, doi:10.7932/NCEDC. The original catalog of LFEs was available in the supplement of Shelly (2017)^7^. The produced LFE catalog in this study is available in Supplementary Data 1.

## Code availability

Most codes used in this study are written in Matlab. Some data pre-processing codes are written in Python. All of them are available upon request to the corresponding author.
