# Supplementary material for: Linking the scaling of tremor and slow slip near Parkfield, CA
Source: Nat Commun. 2022 Oct 3;13:5826. doi: 10.1038/s41467-022-33158-3 (PMC9529943; doi:10.1038/s41467-022-33158-3)
Supplement: Supplementary file 1 — Supplementary information [file 41467_2022_33158_MOESM1_ESM.pdf]

Supplementary information for

Linking the scaling of tremor and slow slip near Parkfield, CA

Hui Huang[1*] and Jessica C. Hawthorne[1]

[1]Department of Earth Sciences, University of Oxford, Oxford, UK

[*]Correspondence to: oldyellow9451@gmail.com

Contents of this file (in order):

Supplementary Figs. 1-10, Supplementary Text (including Supplementary Figs. 11-22)

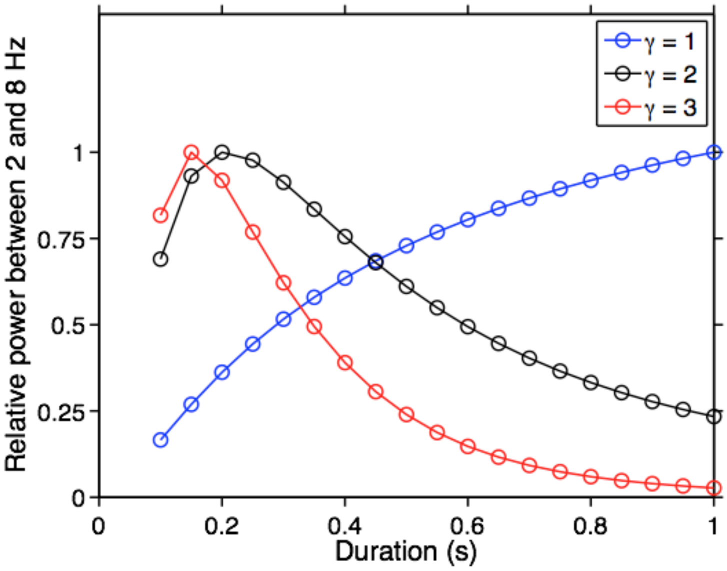

Supplementary Figure 1. The relative mean velocity spectral power between 2 and 8 Hz band, for low frequency earthquakes with different durations (0.1 - 1 sec) that follow a linear moment-duration scaling (see also Figs. 1b and 1c in the main text). For a high-frequency spectral fall-off rate γ = 1 (Ide et al., 2007, see Methods), longer events have larger relative power within 2-8 Hz. For fall-off rates γ >= 2 (Zhang et al., 2011; Supino et al., 2020), the peak occurs at 0.2 sec, suggesting that 0.2-sec duration events have the highest energy within the 2 and 8 Hz band.

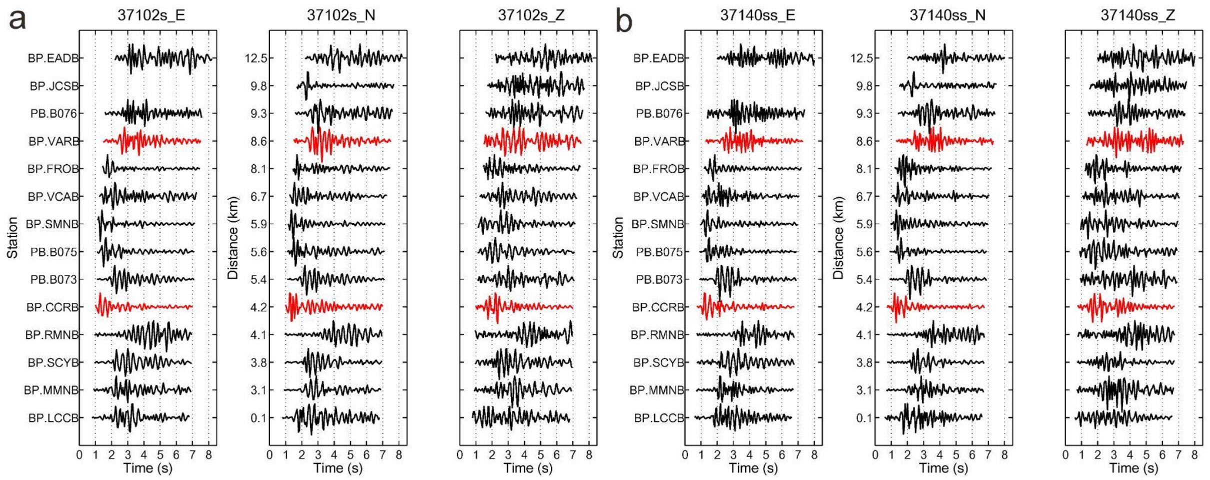

Supplementary Figure 2. The original template waveforms (a: family 37102, b: family 37140) at all components and stations. Red waveforms denote the two independent stations (CCRB and VARB) which are not used in the detection but reserved for validation (see also main text). ‘E’ or ‘N’ are original horizontal components that are close to east or north orientations. E: BP2/SP2/EH2; N: BP3/SP3/EH1; Z: BP1/SP1/EHZ.

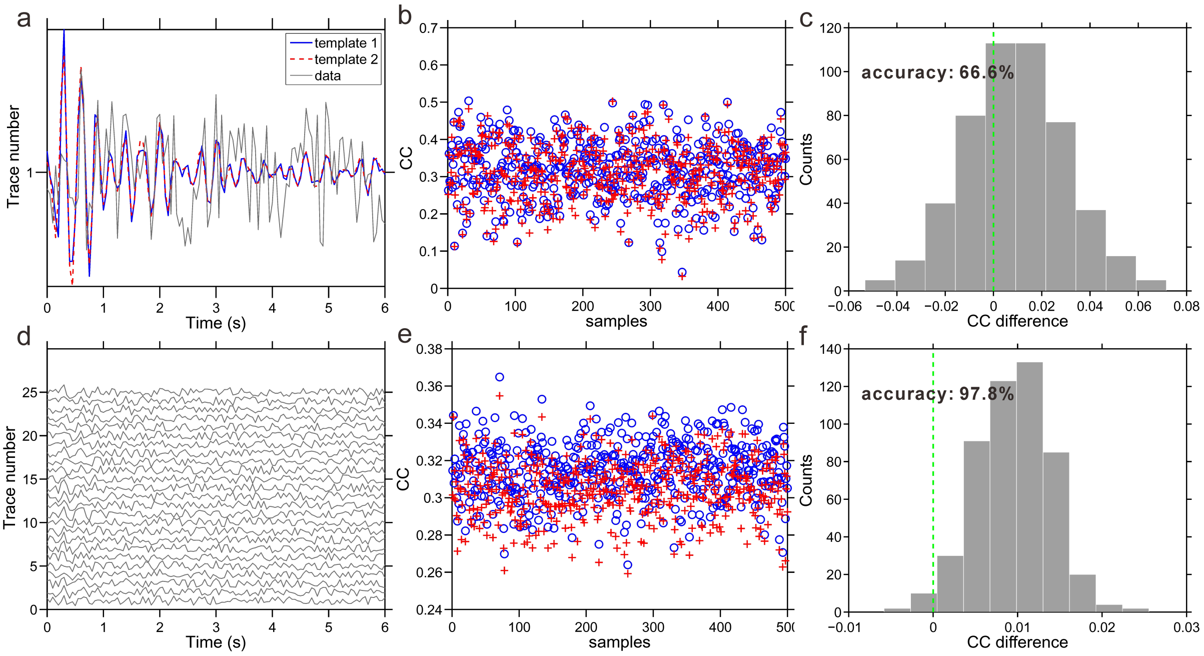

Supplementary Figure 3. Synthetic tests of the duration classification of an event given the noisy data and two highly similar templates. (a) shows the duration classification of an event with one trace (gray line), which is made of random noise plus a 0.2-sec template (E component, station CCRB). Blue and red lines are 0.2- and 0.3-sec templates, respectively. (b) Blue and red symbols denote the cross-correlation coefficients (CC) between 0.2-sec and 0.3-sec templates and the 500 realizations of the data shown in (a), respectively. (c) shows the histogram of the CC differences between the blue and red values shown in (b). A positive difference (to the right of the green dashed line) means that the event's duration is correctly classified. For the single trace, the classification accuracy of durations is ~67%. (d)-(f) are similar to (a)-(c) but for classifying an event's duration with 25 traces. After stacking multiple CC, the classification accuracy of durations increases dramatically to ~98%. See Methods for more details.

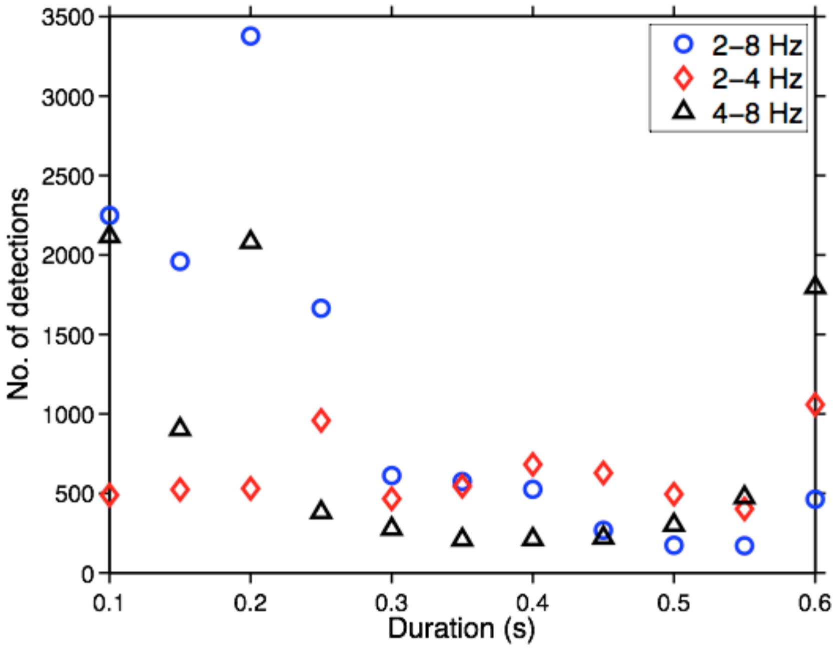

Supplementary Figure 4. The number of detections at different durations under three frequency bands: 2-8 Hz (blue circles), 2-4 Hz (red diamonds) and 4-8 Hz (black triangles).

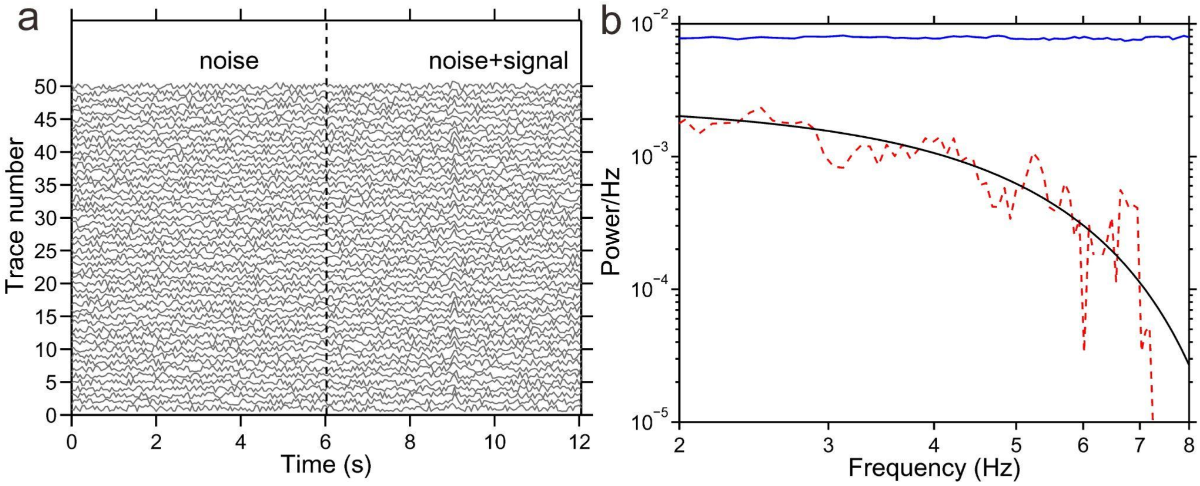

Supplementary Figure 5. Synthetic tests of signal spectral-power recovery given the noisy data. (a) 500 traces of random noises (50 are plotted for clarity) are generated with signals (0.2-sec hann functions) added to the second halves at 9 sec. This is to simulate the event data within a duration group. (b) median noise power (blue), median noise-corrected signal power (red) and ground-truth input signal power (black). They are processed in the same way as the spectra analysis of low frequency earthquakes. See Methods for more details.

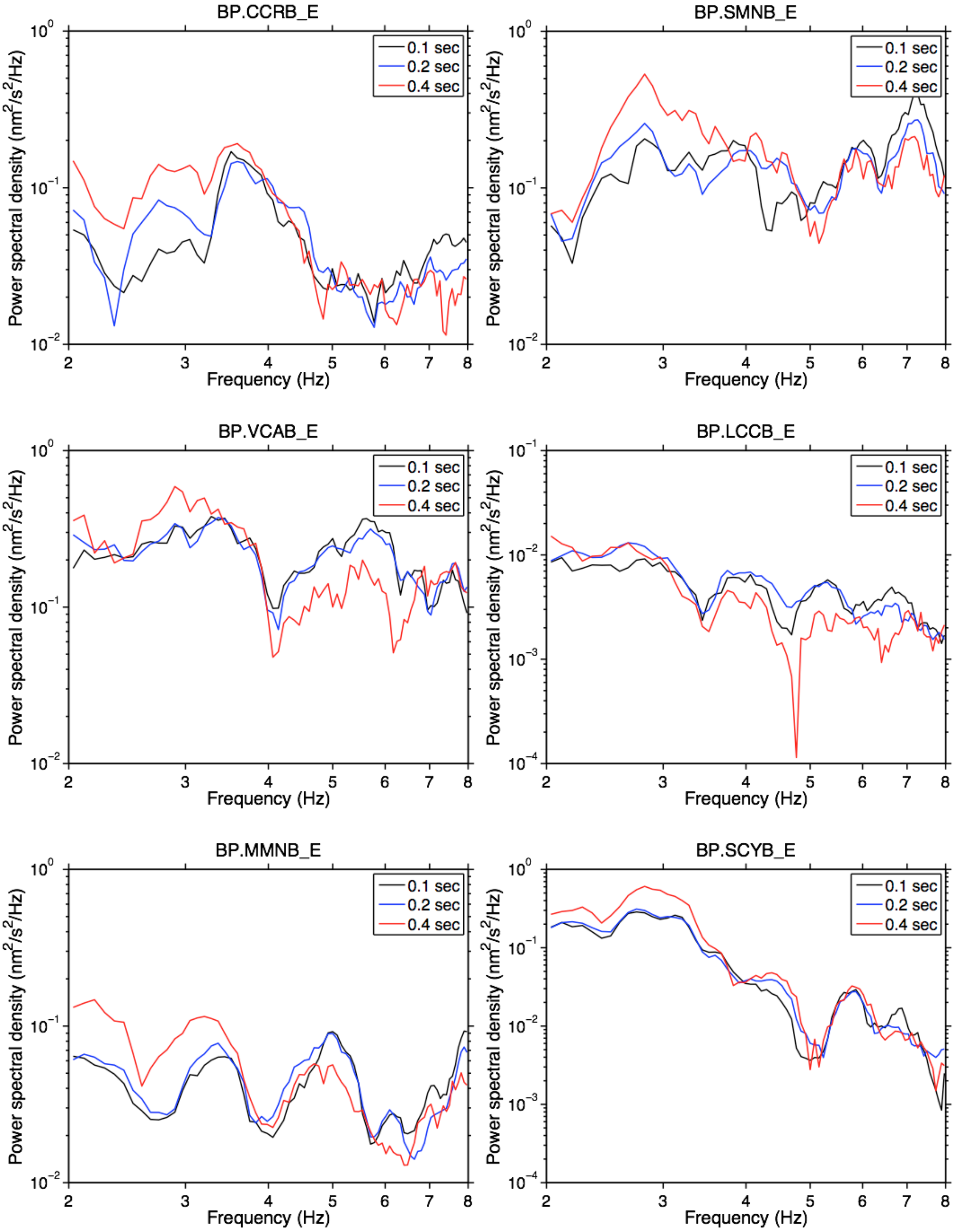

Supplementary Figure 6. Median velocity spectral power for all detection waveforms (family 37102) at different durations: 0.1 sec (black), 0.2 sec (blue) and 0.4 sec (red). Note that the power is obtained by taking the median of individual noise-corrected spectra (see Methods). Powers are calculated with multi-taper methods with three tapers (*NW* = 3). See Supplementary Fig. 2 legend for data channel codes.

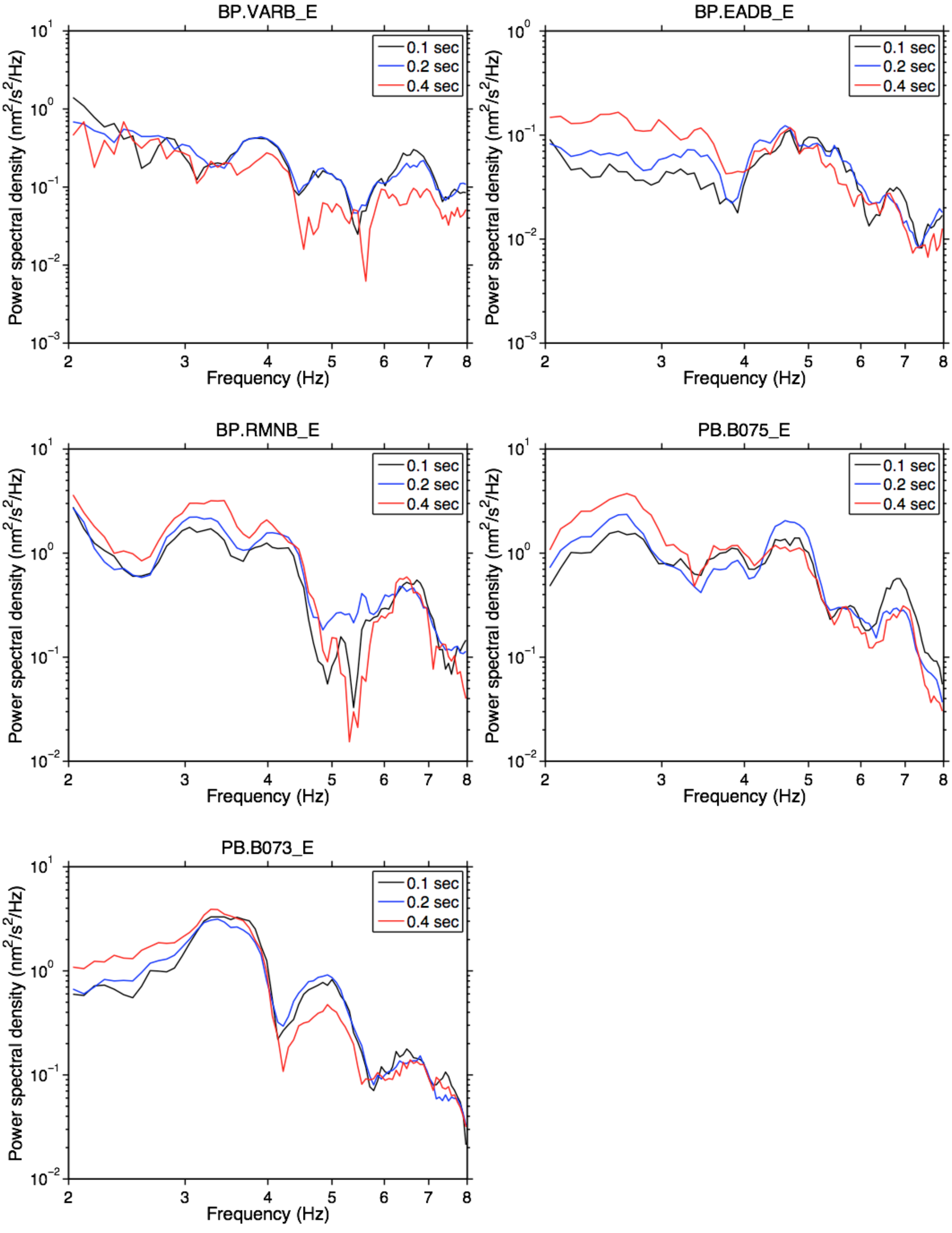

Supplementary Figure 6 (Continued).

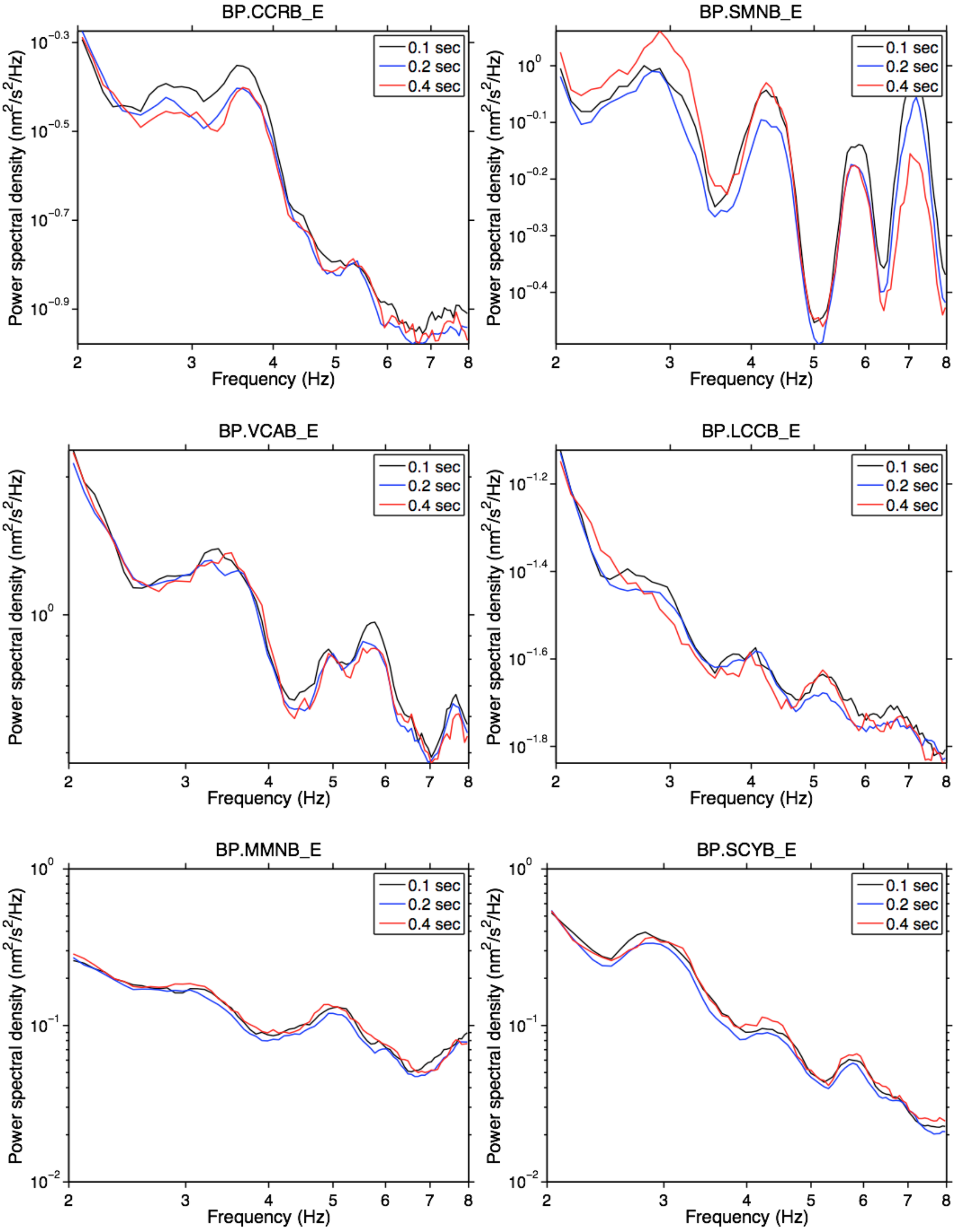

Supplementary Figure 7. Similar to Supplementary Fig. 6 but showing the median noise powers measured on the noise windows 2 sec before the corresponding detection windows (family 37102). These are used to calculate the noise spectral ratios in Fig. 5c in the main text.

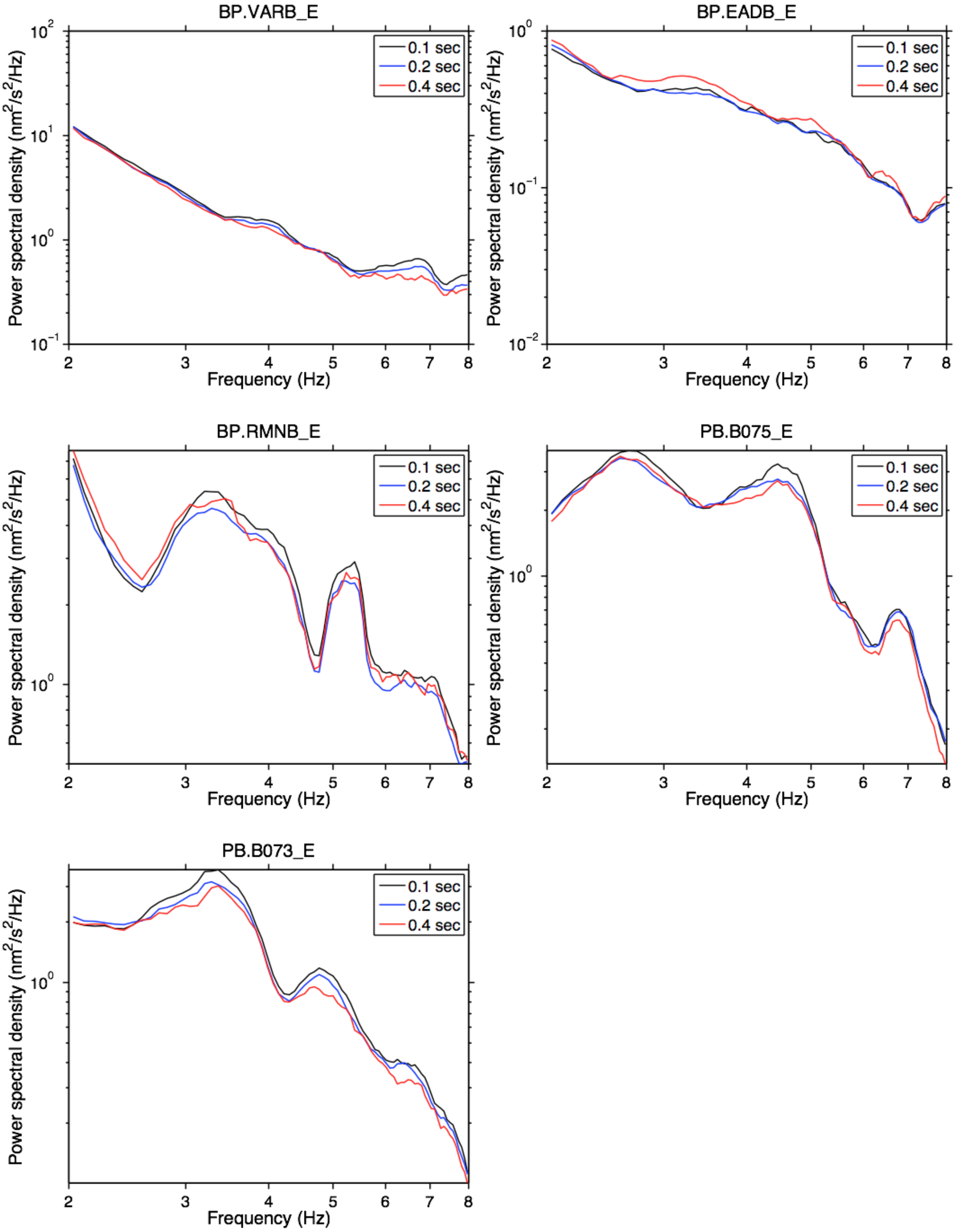

Supplementary Figure 7 (Continued).

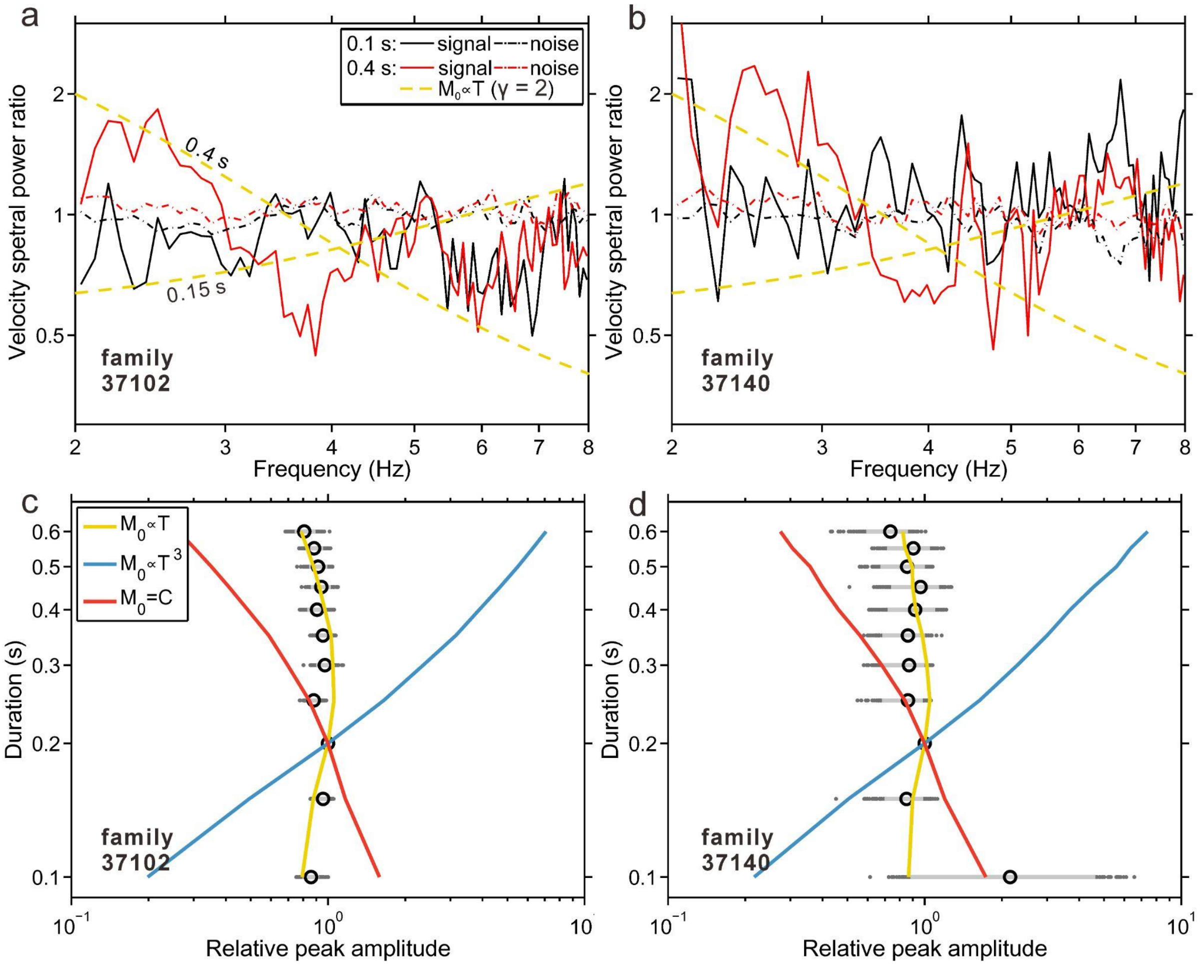

Supplementary Figure 8. Similar to Figs. 5c-5d and 6 in the main text but analyzed for the catalog of low frequency earthquakes detected and classified in the 2-4 Hz band. $M_0 = C$ means that the moment is constant, independent of duration.

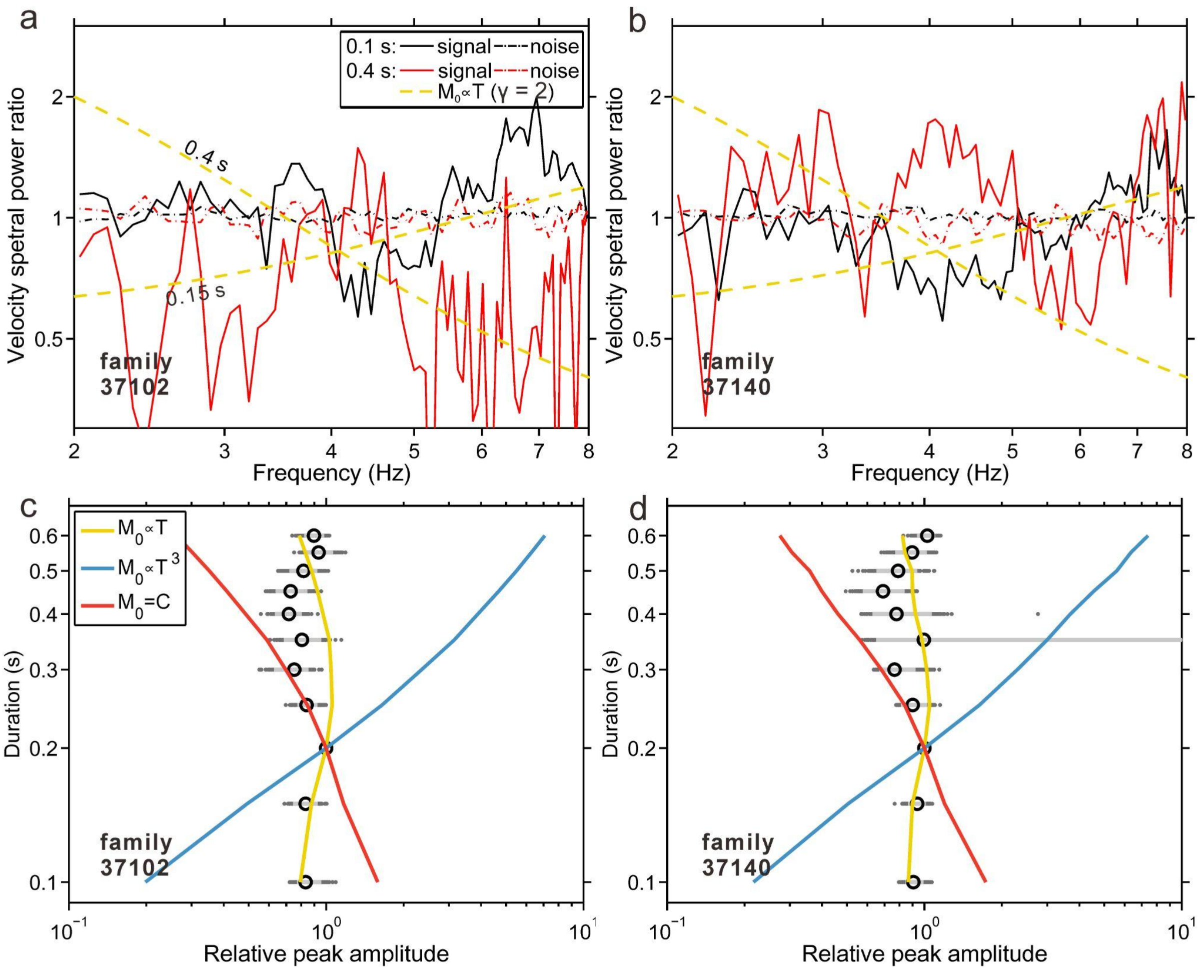

Supplementary Figure 9. Similar to Supplementary Fig. 8 but analyzed for the catalog of low frequency earthquakes detected and classified in the 4-8 Hz band. $M_0 = C$ means that the moment is constant, independent of duration.

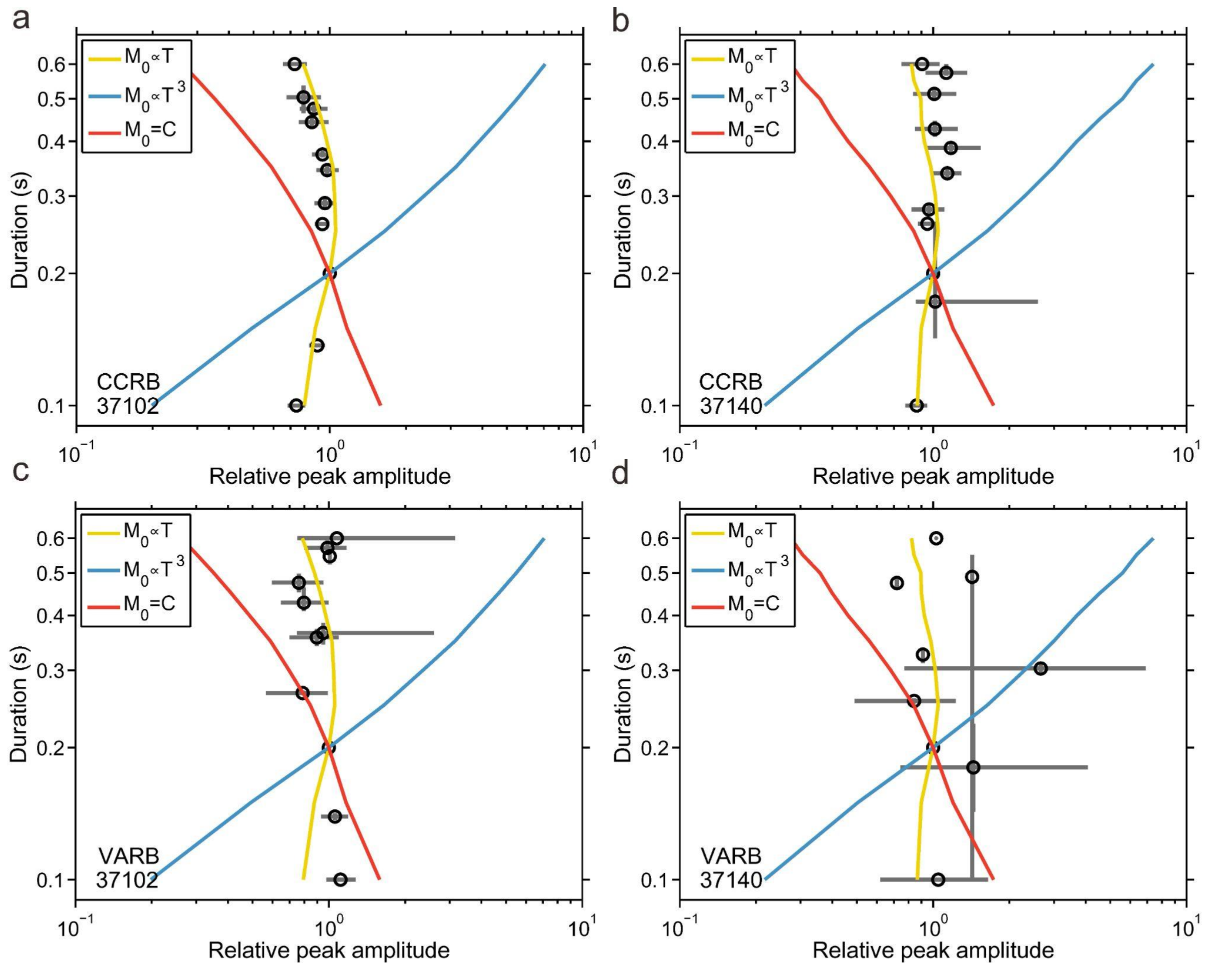

Supplementary Figure 10. Similar to Fig. 6 in the main text but here the amplitudes are calculated at two independent stations. The duration uncertainties (70% confidence) are from Figs. 4e and 4f in the main text. (a)-(b): CCRB; (c)-(d): VARB. $M_0 = C$ means that the moment is constant, independent of duration.

Supplementary Text

Effect of detection bias on the inferred scaling

Section 1: Detectability of various-duration low frequency earthquakes

Here we carry out synthetic tests to understand the detection bias and its effects on moment duration scaling of low frequency earthquakes (LFEs). To understand the varying detection capabilities at different durations, we generate synthetic LFEs with different moments and durations, place them amongst noise, and attempt to detect them.

We generate synthetic LFEs with durations between 0.1 and 0.6 s, using the approach described in the main text and the Methods section. We assign each of these LFEs a relative moment between 10^{-4} and 10^{4}, where a moment of 1 is defined as the moment of the observed 0.2-sec LFE stack. We do not precisely know the absolute moment of that stack; Thomas et al. (2016) suggest a magnitude around 1. But we can rescale the synthetic waveforms at various stations to match the desired relative moment. In this scaling, we account for the amplitudes' dependence on both moment and duration: for the same moment, the LFE's amplitude decreases as duration increases (see Methods and Supplementary Fig. 11). Once we have generated a suite of synthetic LFEs, we add random noise, extracted from real continuous data. For each LFE, we calculate the cross-correlation coefficients between the resulting waveforms and different-duration template waveforms. Then we average the correlation coefficients across different stations and components. An LFE is detected if the average coefficient exceeds the detection threshold, which is the same as that used in the matched-filter detection. Note that the threshold depends on the noise levels on different days, thus we extract noise from data on different days separately. Taking the analysis on 19 July 2012 as an example, whenever an event is detected, it is marked as a black plus sign in Supplementary Fig. 12a. It shows that we miss more small events as the duration increases. That is, the lower detection limit of LFE moments increases with duration. For each duration, we estimate the probabilities of detecting a synthetic LFE as a function of its moment. The probabilities on 19 July 2012 are plotted in Supplementary Fig. 12b, where LFE duration is indexed by color. We repeat the above analysis to all ~850 days separately, to obtain different detection probabilities on different days. Finally, for each duration, we take the median of all

detection probabilities on all days (Supplementary Fig. 12c), which are input into the following detection bias analysis.

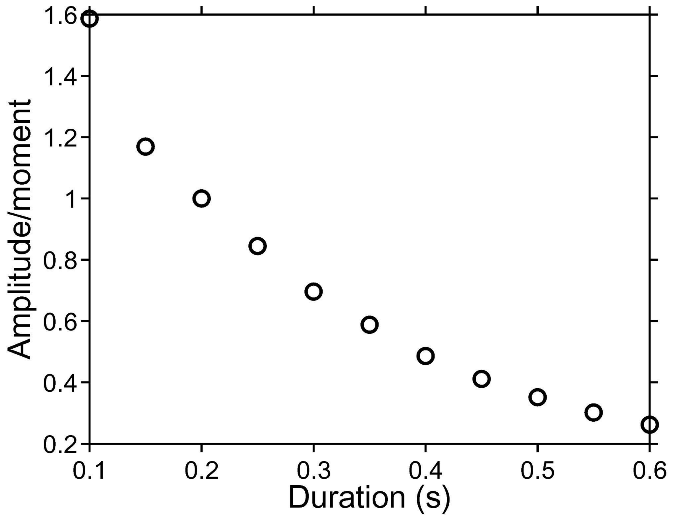

Supplementary Figure 11. The variation of amplitude with duration, estimated from synthetic templates (family 37102) with normalized moments at different durations (see Methods).

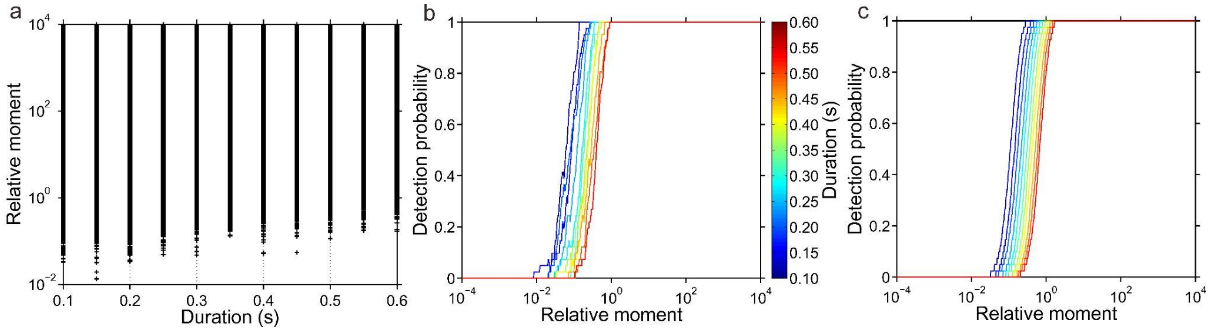

Supplementary Figure 12. Varying detection probabilities of low frequency earthquakes (LFEs) with different durations. (a)-(b) are for detection probability estimated on 19 July 2012. (a) Symbols mark the synthetic events that can be detected under the same threshold used in the matched-filter detection. Moments of the events are relative to the 0.2-sec LFE stack, which is set to 1. (b) The curves show the detection probabilities colored by duration, estimated from the results shown in (a). (c) shows the median detection probability on all ~850 days.

Section 2: Detection bias analysis

Now that we know the detectability of LFEs of various durations and moments, we can investigate how detection bias could affect the moment-duration scaling analysis. We generate several suites of

LFEs which follow different moment-duration scalings, and investigate which moment-duration scalings we would recover given the detection bias.

We begin with three very simple scenarios: collections of LFEs where moment and duration are uncorrelated. In these scenarios, we still assume there are LFEs with a range of durations. But we assume the same moment distributions for LFEs at different durations. We adopt three possible LFE moment distributions from previous studies: a power-law distribution (e.g., Bostock et al., 2015), an exponential distribution (e.g., Chamberlain et al., 2014; Chestler and Creager, 2017), and a log-normal distribution (e.g., Sammis and Bostock, 2021). These input moment probability distributions are illustrated in Supplementary Figs. 13a, 14a, and 15a. We multiply the moment distributions by the detection probabilities for the various durations. This multiplication gives us the distributions of LFE moments we would detect for the three scenarios. They are shown in Supplementary Figs. 13b, 14b, 15b.

Finally, we do the equivalent of LFE stacking. We estimate the mean moment from the corrected moment distribution for each duration. The results are plotted in the dashed lines in Supplementary Figs. 13c, 14c, 15c. For comparison with our observations, we map the mean moments into amplitudes, based on the dependence of amplitude on both moment and duration (see Methods). These amplitudes are plotted as a dashed curve along with our results in Supplementary Figs. 13d, 14d, 15d. It suggests that the no-scaling synthetic scenarios can not match our observations.

Note that, for log-normal and exponential distributions, the input PDF parameters are chosen such that the output mean 0.2-sec moment of the corrected PDF matches the observed 0.2-sec mean moment, which is 1. For the power-law distribution, we test a range of *b* values adopted from previous observations (2-10, e.g. Bostock et al., 2015) and find that the corrected PDFs will always result in a 0.2-sec mean smaller than 1 (*b* value of 5 is shown in Supplementary Fig. 14).

The synthetic tests shown in Supplementary Figs. 13-15 imply that the moment-duration scaling we observe cannot be generated by a suite of LFEs where moment and duration are uncorrelated. So next, we consider four scenarios where the LFE moment distributions vary with duration. We assume that the mean LFE moment scales linearly or cubically with duration. For each duration, we assume that LFE moments follow a log-normal (scenarios 1 and 2, Supplementary Figs. 16 and 17) or exponential (scenarios 3 and 4, Supplementary Figs. 18 and 19) distribution centered around that mean moment. The resulting input moment distributions are plotted in Supplementary Figs. 16a, 17a, 18a, and 19a, where longer LFEs (redder curves) have moment distributions centered around larger values. We again correct for detection bias to determine the moment distributions we would observe (Supplementary Figs. 16b, 17b, 18b and 19b). We estimate the mean moment for each duration (dashed curve in Supplementary Figs. 16c, 17c, 18c and 19c). And we estimate the amplitude that we would observe given that moment: what we would estimate from an LFE stack. We plot these expected amplitudes as a dashed curve in Supplementary Figs. 16d, 17d, 18d, and 19d. Also plotted are the amplitudes of the LFE stacks we observe from the real data (circles). We find that the amplitudes predicted by the cubic moment-duration scaling (Supplementary Figs. 17d and 19d) do not pass through our observations. The linear moment-duration scaling (Supplementary Figs. 16d and 18d), on the other hand, matches our observations well.

Interpreted simply, these results suggest that our observations can be reproduced only by a linear moment-duration scaling. Detection bias does not appear to change the amplitudes of the various-duration stacks significantly enough to shift another scaling towards our observations. We cannot exclude, of course, that our detectability estimates are incorrect. For instance, our synthetic events are perfect matches for our templates, but real long-duration LFEs may be slightly different. However, we note that we do retrieve the same amplitude scalings when we consider detections in different frequency bands; modest differences in detectability thus seem unlikely to significantly change the observed scaling.

For completeness, it may be useful to note that we have made no attempt to constrain the overall LFE moment distribution. That distribution depends not just on the moment distribution at each duration, but also on the relative portion of events at different durations. The varying moment distributions considered here are designed only to simulate the synthetic moment duration scaling.

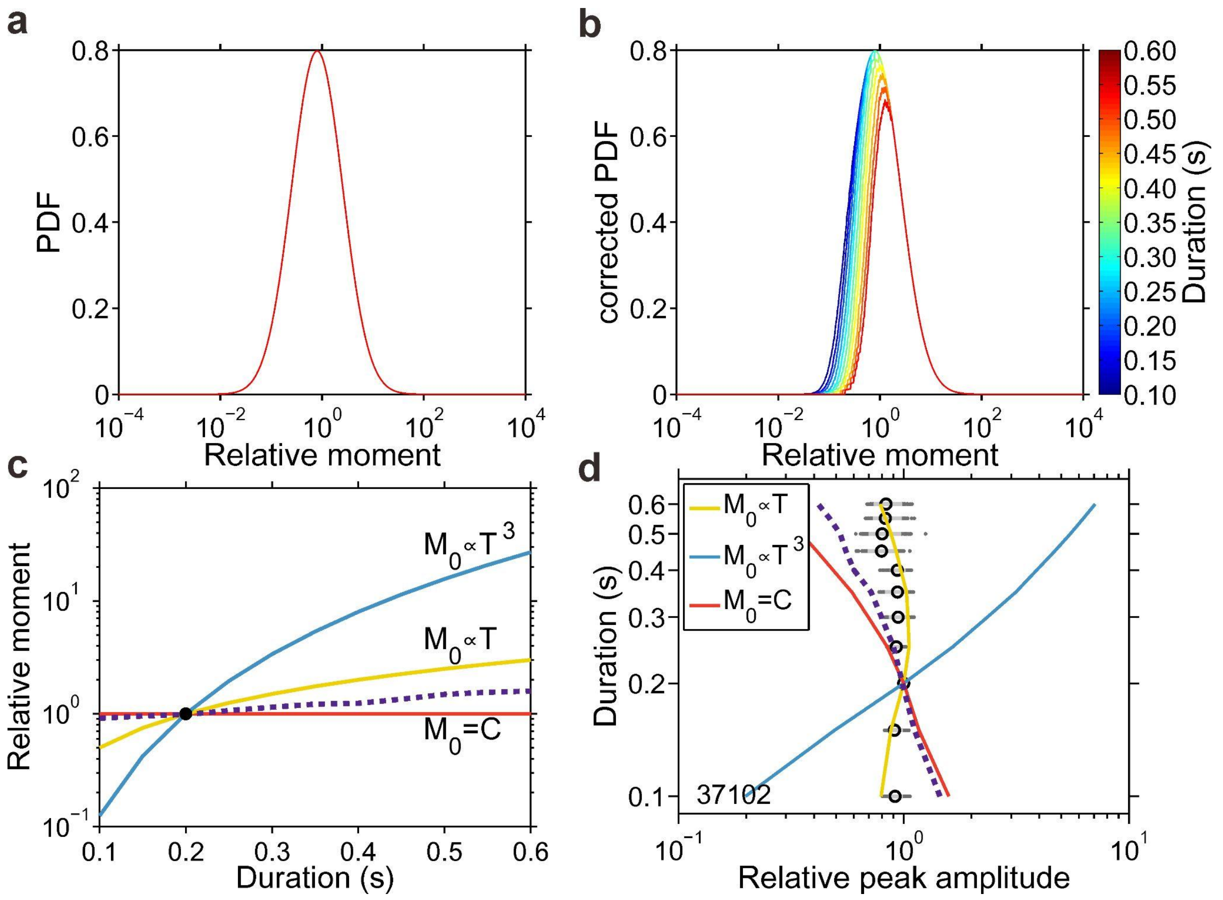

Supplementary Figure 13. Synthetic scenarios of no scaling, where moment and duration are uncorrelated. (a) The suites of low frequency earthquakes at different durations are assumed to follow the same log-normal probability density function (PDF) with a mean of 0.8 and standard deviation of 0.5. (b) The corrected PDFs at different durations are generated by multiplying (a) with corresponding detection probabilities shown in Supplementary Fig. 12c. (c) The dashed lines show the estimated mean moment as a function of duration, from the corrected PDFs shown in (b). (d) The dashed line shows the amplitudes converted from the moments (dashed line in (c)). See Methods for more details on mapping moment into amplitude. $M_0 = C$ means that the moment is constant, independent of duration.

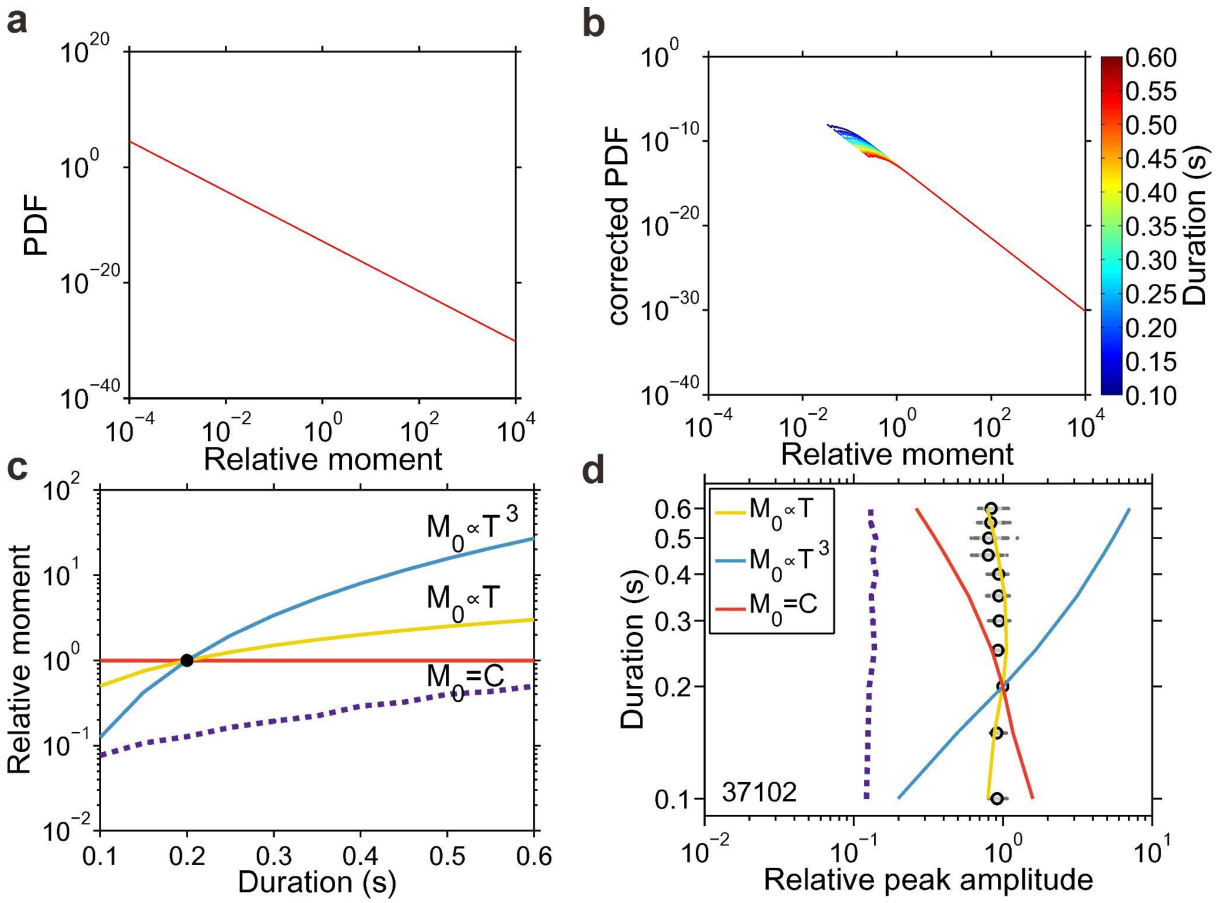

Supplementary Figure 14. Similar to Supplementary Fig. 13 but for various-duration low frequency earthquakes following the same power-law moment distribution with an equivalent *b* value of 5 (which defines the slope in (a)). $PDF(M_0) = \beta M_{0thre}^{\beta}/M_0^{\beta+1}$, where $\beta = b(2/3)$, M_{0thre} is the lower bound of the distribution, which is set to 10^{-4}.

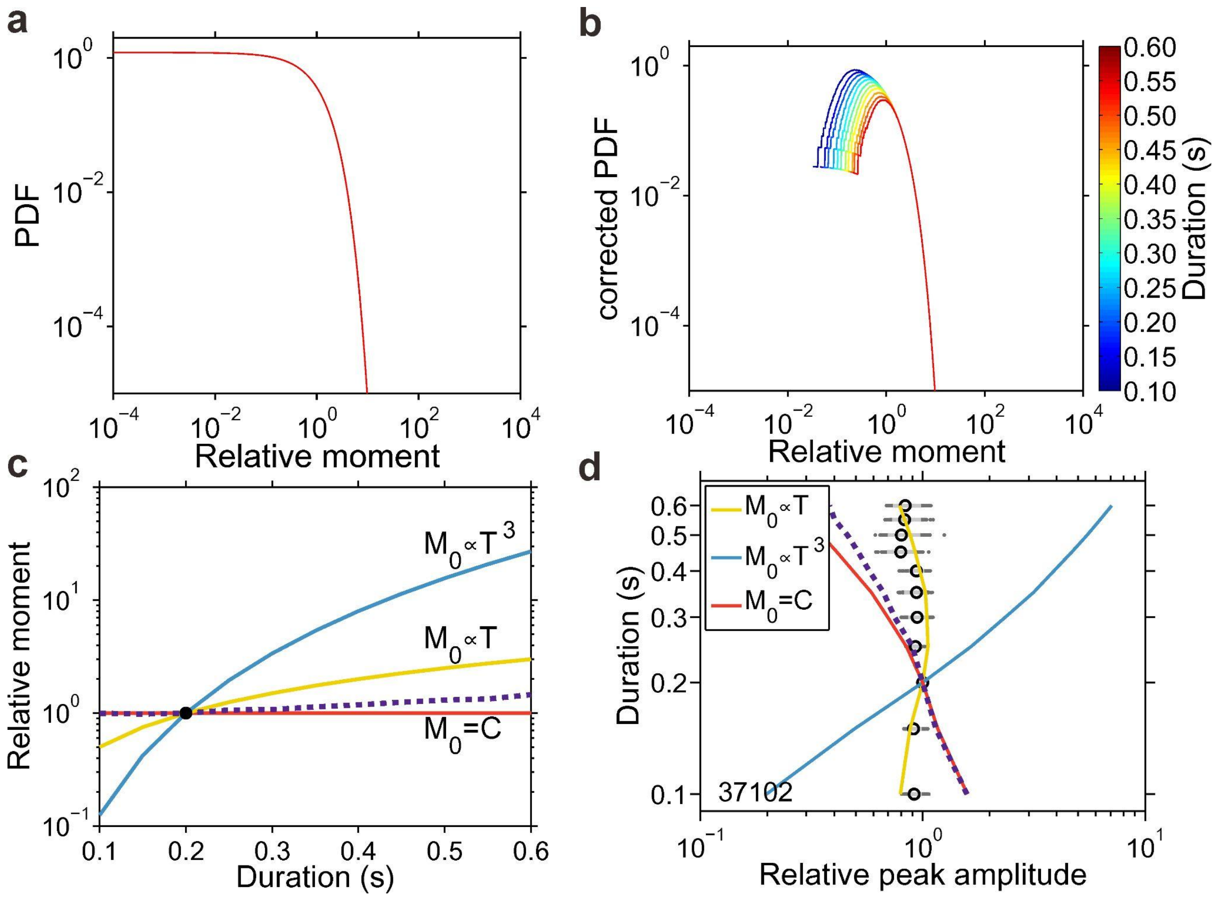

Supplementary Figure 15. Similar to Supplementary Fig. 13 but for various-duration low frequency earthquakes following the same exponential moment distribution with λ of 1.2. $PDF(M_0) = \lambda exp(-\lambda M_0)$, which has a mean of $1/\lambda$.

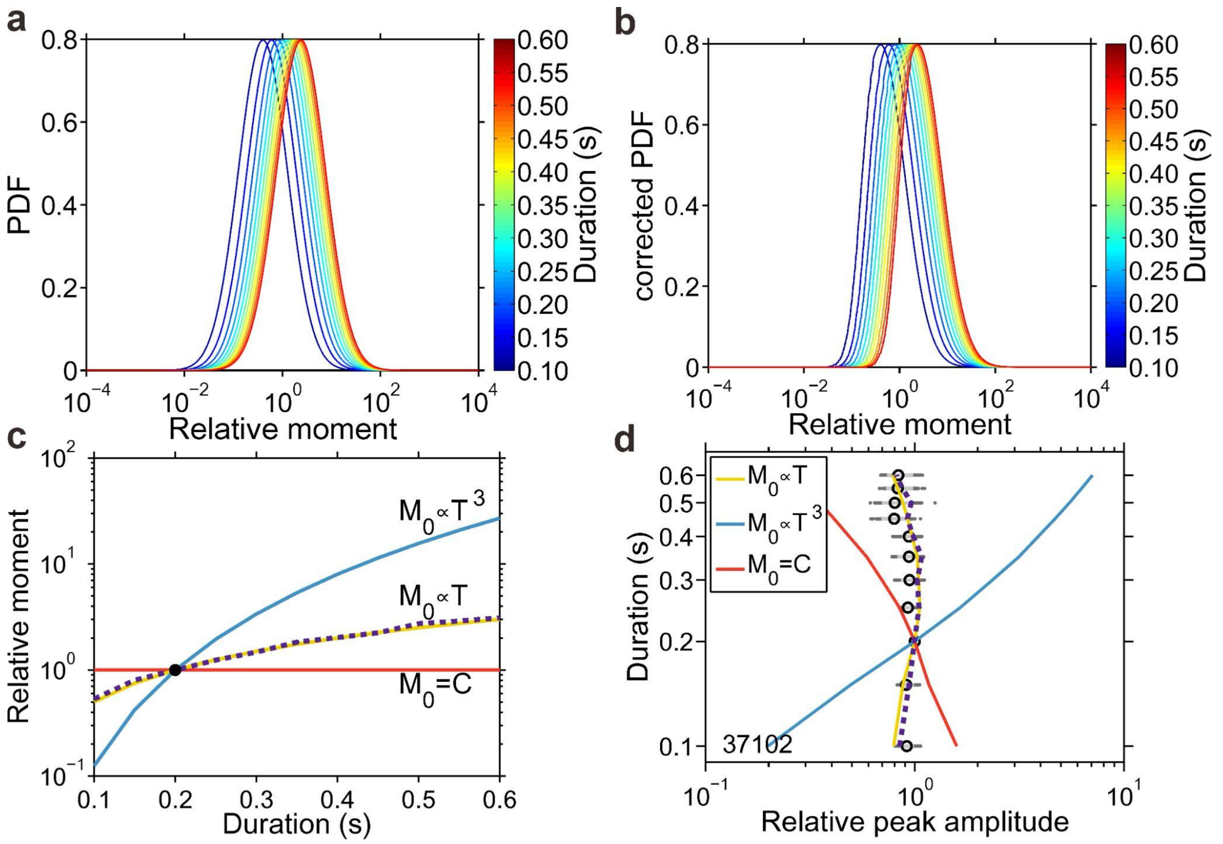

Supplementary Figure 16. Synthetic scenarios of moment duration scaling where moment and duration are correlated. (a). Varying log-normal moment distributions are assumed for low frequency earthquakes with the corresponding mean moment and duration following a linear moment-duration scaling. The probability density function (PDF) at 0.2 sec is the same as that shown in Supplementary Fig. 13a. (b)-(d) are similar to those shown in Supplementary Figs. 13b-13d.

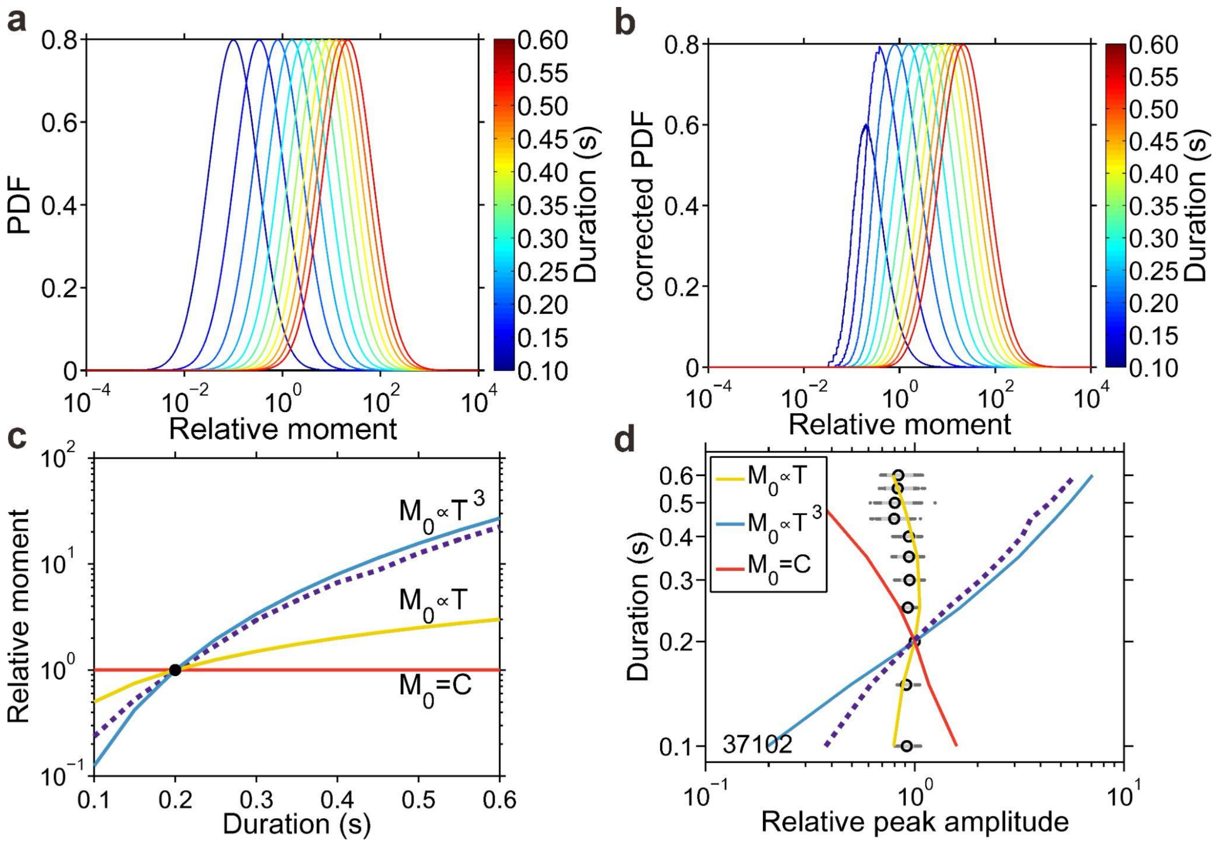

Supplementary Figure 17. Similar to Supplementary Fig. 16 but for an input of moment duration cubed scaling.

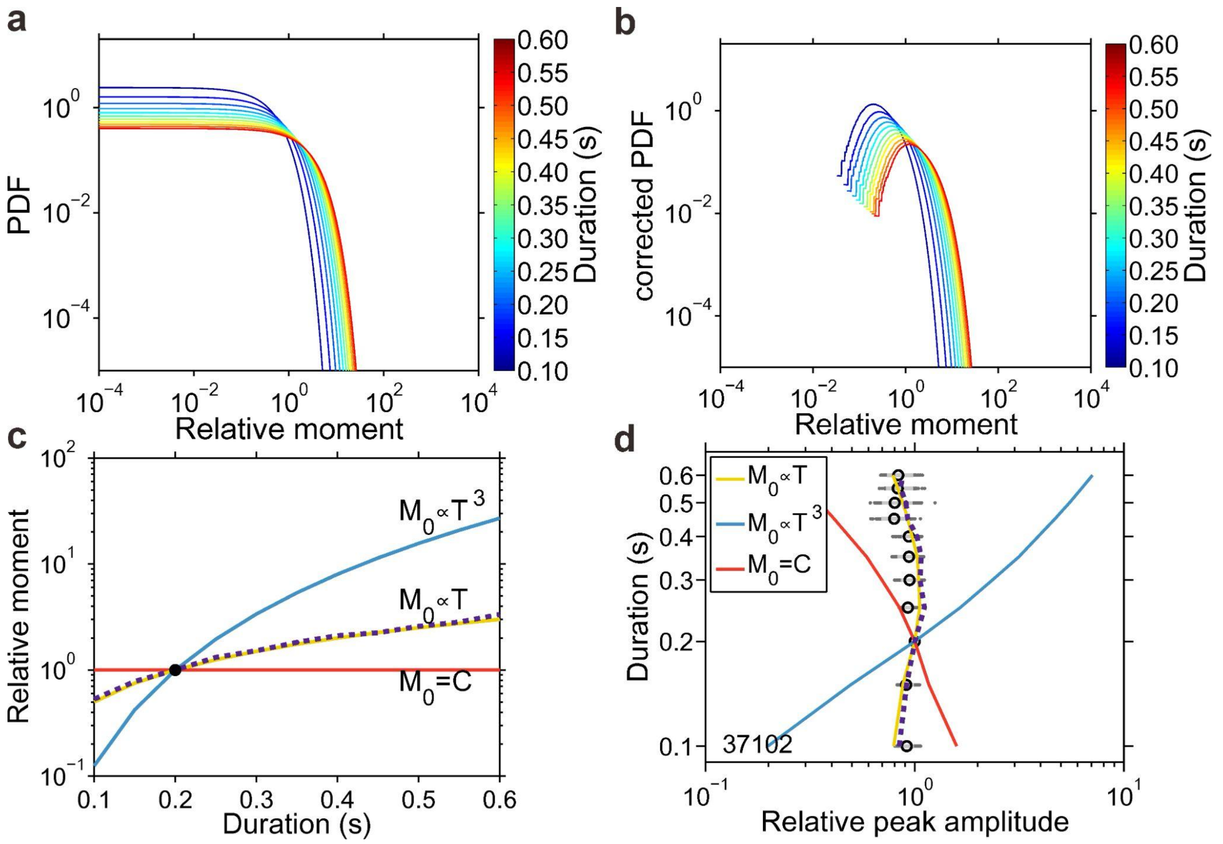

Supplementary Figure 18. (a). Varying exponential moment distributions are assumed for low frequency earthquakes with the corresponding mean moment and duration following a linear moment duration scaling. The probability density function (PDF) at 0.2 sec is the same as that shown in Supplementary Fig. 15a. (b)-(d) are similar to those shown in Supplementary Figs. 13b-13d.

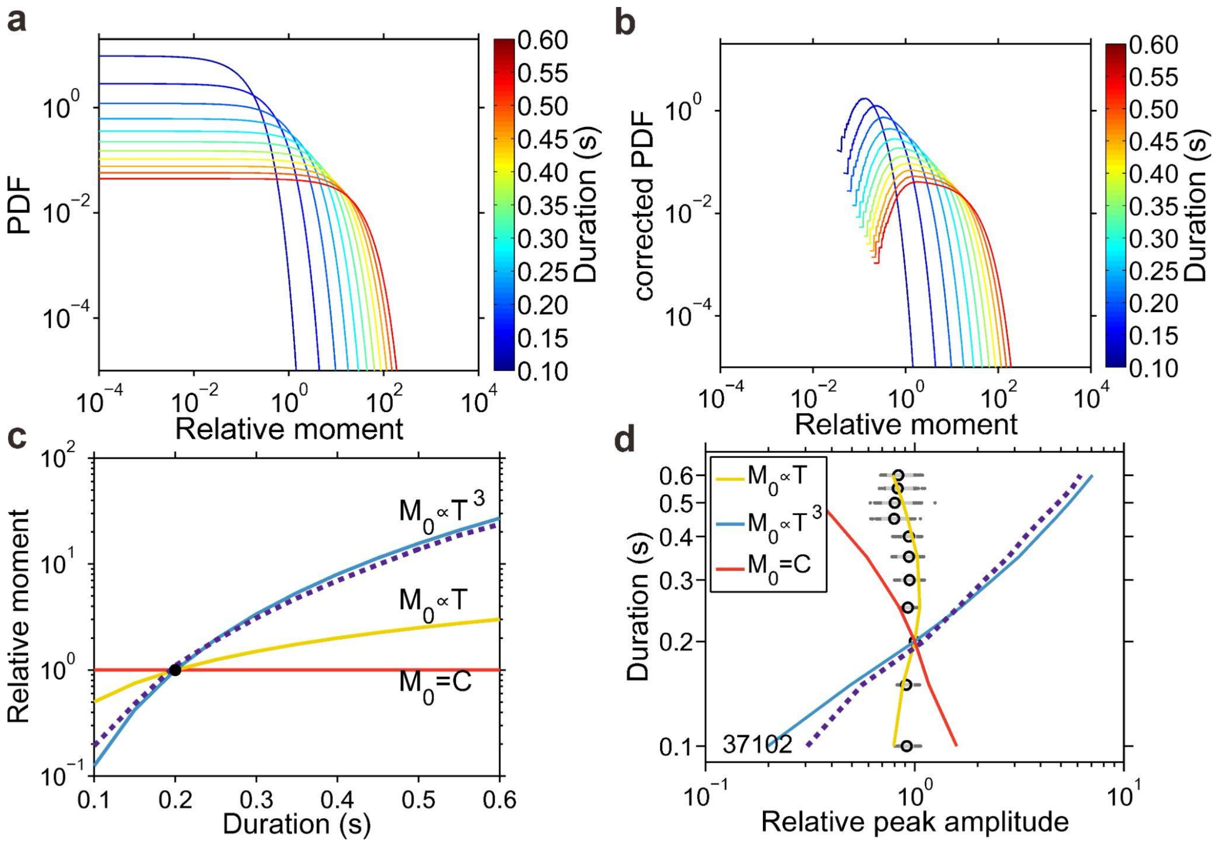

Supplementary Figure 19. Similar to Supplementary Fig. 18 but for an input of moment duration cubed scaling.

Effect of data uncertainties on moment duration scaling

Previous studies measure moments and corner frequencies for individual LFEs, then stack the corner frequencies within moment bins (e.g., Farge et al., 2020; Supino et al., 2020). This is based on the assumption that moment uncertainties are smaller than corner frequency uncertainties. We add a note here that this strategy might lead to bias if the relative uncertainties of moments and corner frequencies are not well understood.

We add one extra analysis of the dataset produced by Supino et al. (2020), for LFEs in the Nankai subduction zone. We find that binning by corner frequency can lead to a scaling close to $M_0 \propto T$ while binning by moment leads to a $M_0 \propto T^3$ scaling (Supplementary Fig. 20). To understand this difference, we carry out two synthetic tests by generating data points of moments and corner frequencies, following $M_0 \propto T$ or $M_0 \propto T^3$ scaling relations. For the $M_0 \propto T$ dataset (Supplementary Fig. 21a), we

fix the corner frequencies but add random errors to the moments (Supplementary Figs. 21b, 21c). In contrast, for the $M_0 \propto T^3$ dataset (Supplementary Fig. 22a), we fix the moments but add random errors to the corner frequencies (Supplementary Figs. 22b, 22c). We find that for both cases, binning by moment and corner frequency result in different scalings. Binning along the variable with zero uncertainties can correctly recover the input scaling (Supplementary Figs. 21b, 22c), but binning along the variable with non-zero uncertainties will result in a bias (Supplementary Figs. 21c, 22b). The principle component analysis (PCA), which is equivalent to total least squares along both axes, also gives biased results in these cases. In contrast, the Deming regression (orthogonal regression), which takes account of the relative uncertainties of moments and corner frequencies, correctly recovers the input scalings if we input the correct variance ratio into the regression (Supplementary Figs. 21b, 22b). This indicates that it is necessary to take into account the relative uncertainties of moments and corner frequencies in the regression. Further analysis is needed to obtain the robust variance ratio between moment and corner frequency in real measurements.

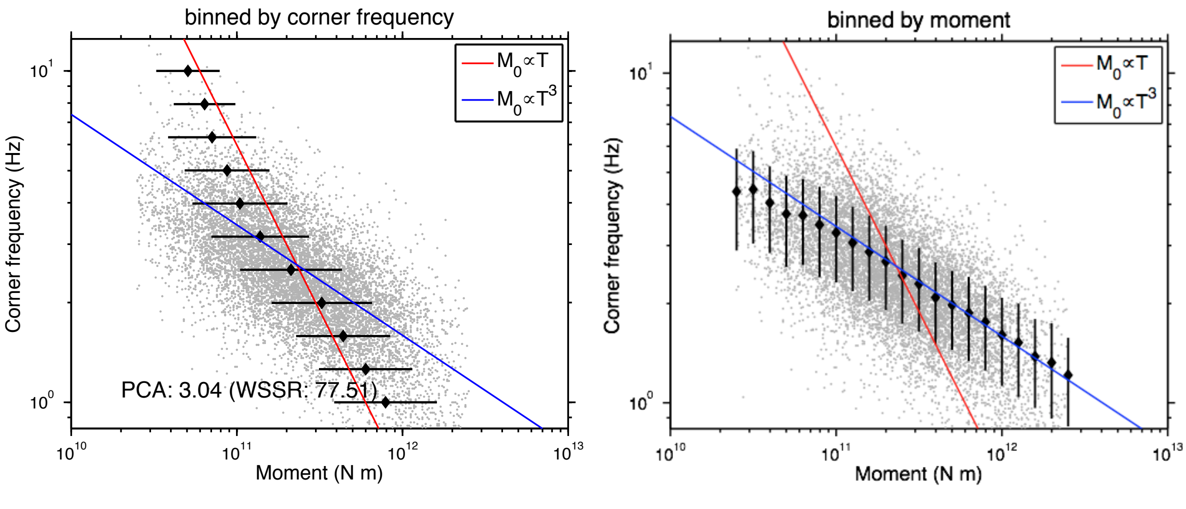

Supplementary Figure 20. Different scaling results when binning observations by corner frequency (left) and moment (right), using the data by Supino et al. (2020). The principle component analysis (PCA) gives a scaling exponent of 3.04. The weighted sum of squared residuals (WSSR) is listed in brackets.

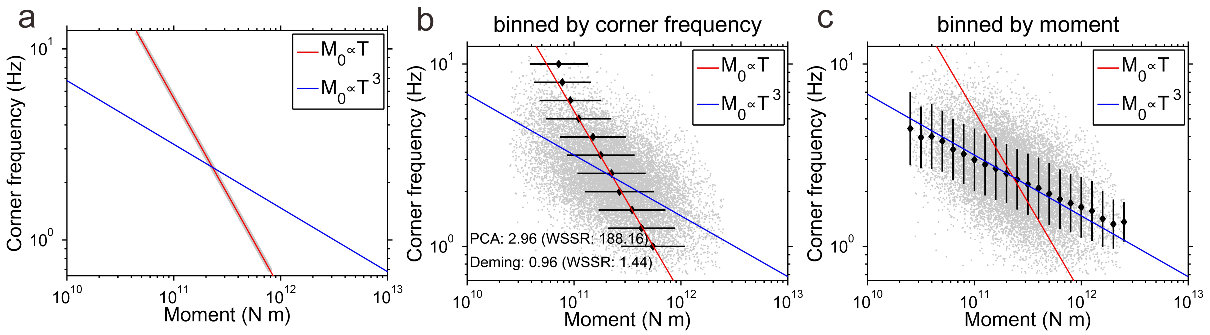

Supplementary Figure 21. (a) Synthetic data points (gray dots) following a linear moment-duration scaling (red line). (b) Synthetic data from (a) are randomly perturbed along the moment axis, while the corner frequencies of the data points are kept unchanged. Note that binning by corner frequency (b) and by moment (c) give different scaling results. The principle component analysis (PCA) results in a large bias of the input exponent while the Deming regression recovers the input exponent. The weighted sum of squared residuals (WSSR) is listed in brackets.

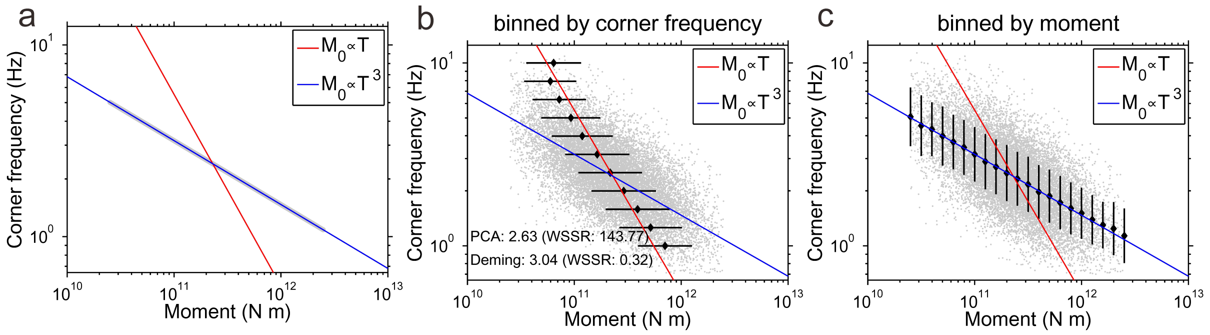

Supplementary Figure 22. (a) Synthetic data points (gray dots) following a moment duration cubed scaling (blue line). Synthetic data from (a) are randomly perturbed along the corner frequency axis, while the moments of the data points are kept unchanged. Note that binning by corner frequency (b) and by moment (c) give different scaling results. The principle component analysis (PCA) results in a slight bias of the input exponent while the Deming regression recovers the input exponent. The weighted sum of squared residuals (WSSR) is listed in brackets.

References

1. Ide, S., Beroza, G. C., Shelly, D. R., & Uchide, T. (2007). A scaling law for slow earthquakes. *Nature*, *447*(7140), 76-79.

2. Farge, G., Shapiro, N. M., & Frank, W. B. (2020). Moment-duration scaling of low-frequency earthquakes in Guerrero, Mexico. *Journal of Geophysical Research: Solid Earth*, *125*(8), e2019JB019099.

3. Supino, M., Poiata, N., Festa, G., Vilotte, J. P., Satriano, C., & Obara, K. (2020). Self-similarity of low-frequency earthquakes. *Scientific reports*, *10*(1), 1-9.

4. Zhang, J., Gerstoft, P., Shearer, P. M., Yao, H., Vidale, J. E., Houston, H., & Ghosh, A. (2011). Cascadia tremor spectra: Low corner frequencies and earthquake-like high-frequency falloff. *Geochemistry, Geophysics, Geosystems*, *12*(10).

5. Bostock, M. G., Thomas, A. M., Savard, G., Chuang, L., & Rubin, A. M. (2015). Magnitudes and moment-duration scaling of low-frequency earthquakes beneath southern Vancouver Island. *Journal of Geophysical Research: Solid Earth*, *120*(9), 6329-6350.

6. Chamberlain, C. J., Shelly, D. R., Townend, J., & Stern, T. A. (2014). Low-frequency earthquakes reveal punctuated slow slip on the deep extent of the Alpine fault, New Zealand. *Geochemistry, Geophysics, Geosystems*, *15*(7), 2984-2999.

7. Chestler, S. R., & Creager, K. C. (2017). Evidence for a scale-limited low-frequency earthquake source process. *Journal of Geophysical Research: Solid Earth*, *122*(4), 3099-3114.

8. Sammis, C. G., & Bostock, M. G. (2021). A granular jamming model for low-frequency earthquakes. *Journal of Geophysical Research: Solid Earth*, *126*(7), e2021JB021963.
