# Supplementary material for: Linking the scaling of tremor and slow slip near Parkfield, CA
Source: Nat Commun. 2022 Oct 3;13:5826. doi: 10.1038/s41467-022-33158-3 (PMC9529943; doi:10.1038/s41467-022-33158-3)
Supplement: Supplementary file 2 — Description of Additional Supplementary Information [file 41467_2022_33158_MOESM2_ESM.pdf]

Description of Additional Supplementary Information

Title: Supplementary Data 1

Description: Low frequency earthquake catalogue with durations near Parkfield
